# Biologic canine and human intervertebral disc repair by notochordal cell-derived matrix: from bench towards bedside

**DOI:** 10.18632/oncotarget.25476

**Published:** 2018-05-29

**Authors:** Frances C. Bach, Anna R. Tellegen, Martijn Beukers, Alberto Miranda-Bedate, Michelle Teunissen, Willem A.M. de Jong, Stefan A.H. de Vries, Laura B. Creemers, Karin Benz, Björn P. Meij, Keita Ito, Marianna A. Tryfonidou

**Affiliations:** ^1^ Department of Clinical Sciences of Companion Animals, Faculty of Veterinary Medicine, Utrecht University, Utrecht, 3584 CM, The Netherlands; ^2^ Orthopaedic Biomechanics, Department of Biomedical Engineering, Eindhoven University of Technology, Eindhoven, MB 5600, The Netherlands; ^3^ Department of Orthopedics, University Medical Centre Utrecht, Utrecht, 3584 CX, The Netherlands; ^4^ TETEC AG, Reutlingen 72770, Germany

**Keywords:** intervertebral disc, regenerative medicine, notochordal cells, canine, human

## Abstract

The socioeconomic burden of chronic back pain related to intervertebral disc (IVD) disease is high and current treatments are only symptomatic. Minimally invasive strategies that promote biological IVD repair should address this unmet need. Notochordal cells (NCs) are replaced by chondrocyte-like cells (CLCs) during IVD maturation and degeneration. The regenerative potential of NC-secreted substances on CLCs and mesenchymal stromal cells (MSCs) has already been demonstrated. However, identification of these substances remains elusive. Innovatively, this study exploits the regenerative NC potential by using healthy porcine NC-derived matrix (NCM) and employs the dog as a clinically relevant translational model. NCM increased the glycosaminoglycan and DNA content of human and canine CLC aggregates and facilitated chondrogenic differentiation of canine MSCs *in vitro*. Based on these results, NCM, MSCs and NCM+MSCs were injected in mildly (spontaneously) and moderately (induced) degenerated canine IVDs *in vivo* and, after six months of treatment, were analyzed. NCM injected in moderately (induced) degenerated canine IVDs exerted beneficial effects at the macroscopic and MRI level, induced collagen type II-rich extracellular matrix production, improved the disc height, and ameliorated local inflammation. MSCs exerted no (additive) effects. In conclusion, NCM induced *in vivo* regenerative effects on degenerated canine IVDs. NCM may, comparable to demineralized bone matrix in bone regeneration, serve as ‘instructive matrix’, by locally releasing growth factors and facilitating tissue repair. Therefore, intradiscal NCM injection could be a promising regenerative treatment for IVD disease, circumventing the cumbersome identification of bioactive NC-secreted substances.

## INTRODUCTION

Over 80% of the human population experiences low back pain (LBP) at least once in their life, with severe socioeconomic consequences [1]. Degeneration of the intervertebral disc (IVD) is a common cause of chronic LBP [2]. Current treatments for LBP due to IVD degeneration mainly aim at symptom reduction or IVD replacement and are not without their inherent limitations. Therefore, LBP should be addressed at an earlier stage by minimally invasive strategies that induce definitive long-term biological IVD repair.

The IVD transmits loads and provides flexibility to the spine. The healthy IVD consists of a gelatinous nucleus pulposus (NP), surrounded by a fibrous annulus fibrosus (AF) and cartilaginous end plates (EPs). During IVD degeneration, the glycosaminoglycan (GAG) content of the NP decreases and collagen type II is replaced by collagen type I, resulting in a more rigid NP tissue that is not able to sustain compressive loads anymore. Since the IVD cannot adequately repair its matrix, the IVD weakens and is further damaged by physiologic loading [3]. During IVD maturation in humans, a transition in NP cell phenotype takes place from large, vacuolated notochordal cells (NCs) to smaller, non-vacuolated chondrocyte-like cells (CLCs) [4]. NC loss in certain species (*e.g.* chondrodystrophic dogs) coincides with the onset of degenerative IVD changes [3]. Moreover, the regenerative potential of NCs has already been demonstrated on CLCs [5–7], mesenchymal stromal cells (MSCs) [8–10], and NP tissue explants [11] *in vitro*, and in rat IVDs *in vivo* [12]. Altogether, this indicates that NCs can play a role in maintaining healthy NP tissue. Therefore, NCs are a promising target for regenerative and/or symptom modifying therapies for IVD disease.

Despite the current focus on the NC secretome [12–14], we here take an innovative approach, by using the entire NC-derived matrix (NCM) from healthy NC-rich NP tissue, as a first step towards clinical translation. NCM may act rather comparable to demineralized bone matrix (DBM), derived from bovine or human donors (*e.g.* Bio-Oss^®^, DBX^®^). DMB is known to contain ECM and growth factor components native to bone and is currently successfully employed in clinical practice to accelerate bone healing [15]. Therefore, the first aim of this study was to determine the (regenerative) effects of NCM on CLCs from degenerated IVDs *in vitro* and injected NCM in degenerated IVDs *in vivo*. For this purpose, we employed porcine NCM given that we have previously shown that porcine NCs exert the strongest anabolic effects on CLCs derived from human and canine degenerated IVDs over human and canine NCs [5]. Since cell viability is impaired in the degenerated IVD [16], NCM alone may not be sufficient. In this respect, intradiscal MSC transplantation is considered a promising regeneration strategy, currently explored in clinical trials (NCT01290367, NCT02412735) [17]. Therefore, the second aim of this study was to determine the effects of NCM combined with MSCs on degenerated canine IVDs *in vivo*. The dog served herein as a translational model, since they experience LBP due to IVD degeneration with similar characteristics as humans [18].

## RESULTS

### NCM increased DNA content, GAG and collagen deposition in CLC micro-aggregates *in vitro*

**Figure 1:**
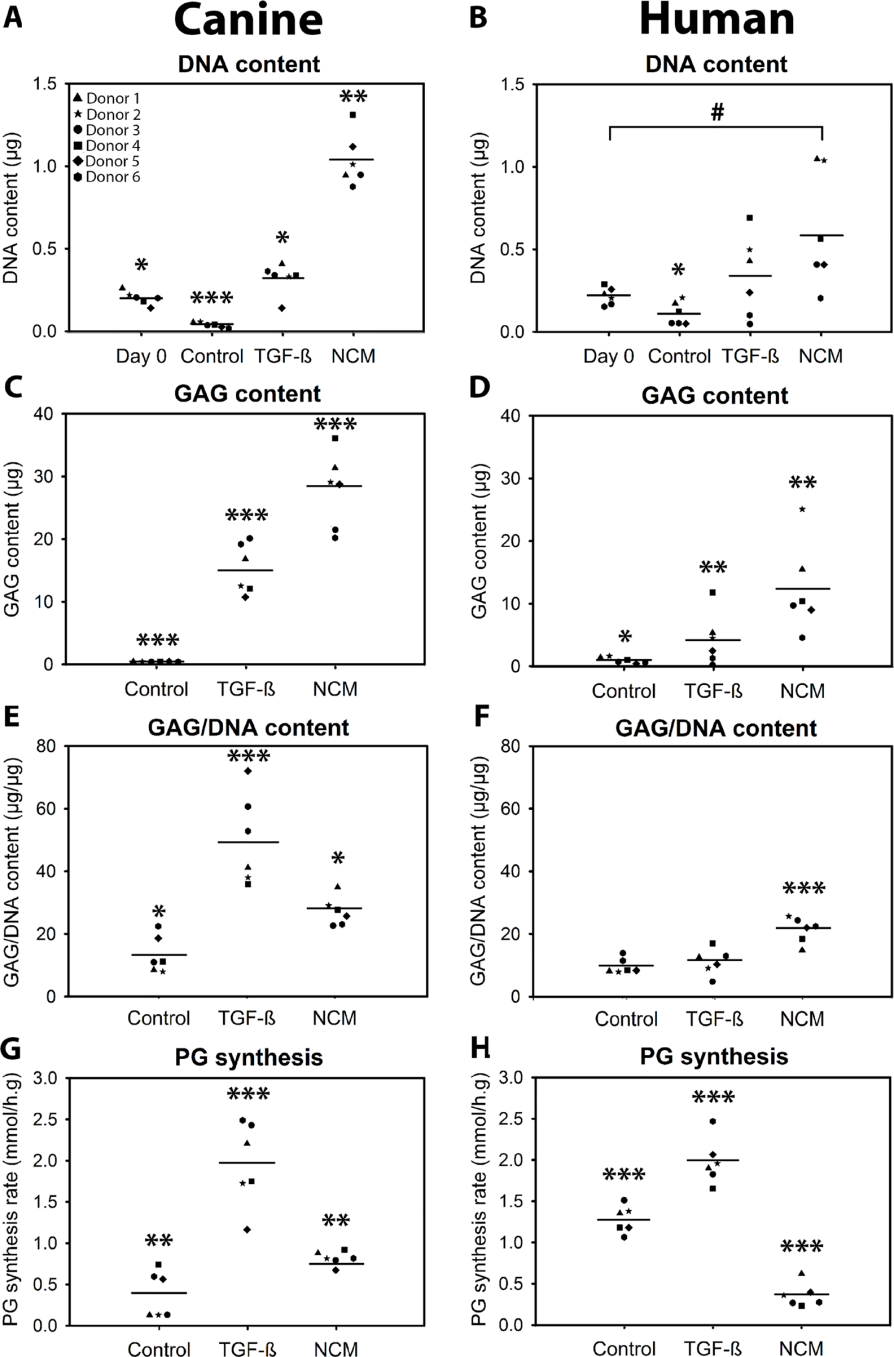
Notochordal cell-derived matrix (NCM) increases the DNA (**A**, **B**), glycosaminoglycan (GAG; **C**, **D**) and GAG/DNA (**E**, **F**) content of chondrocyte-like cell (CLC) micro-aggregates. Average of two samples per donor with depicted mean values. CLC micro-aggregates of 35,000 cells were cultured in basal culture medium (negative control), supplemented with 10 ng/mL TGF-β_1_ (positive control), or 10 mg/mL NCM for 28 days. ^35^SO_4_^2-^ incorporation (proteoglycan (PG) synthesis rate) was determined at day 7 (**G**, **H**). ^*,**,***^p < 0.05, *p* < 0.01, and *p* < 0.001, respectively, for the indicated condition versus all other conditions; ^#^*p* < 0.05 for the two indicated conditions by the horizontal bar; *n* = 6 per species, in duplicates. ANOVA (normally distributed data), Kruskal Wallis/Mann–Whitney U (non-parametric data), and Benjamini & Hochberg tests were performed.

**Figure 2:**
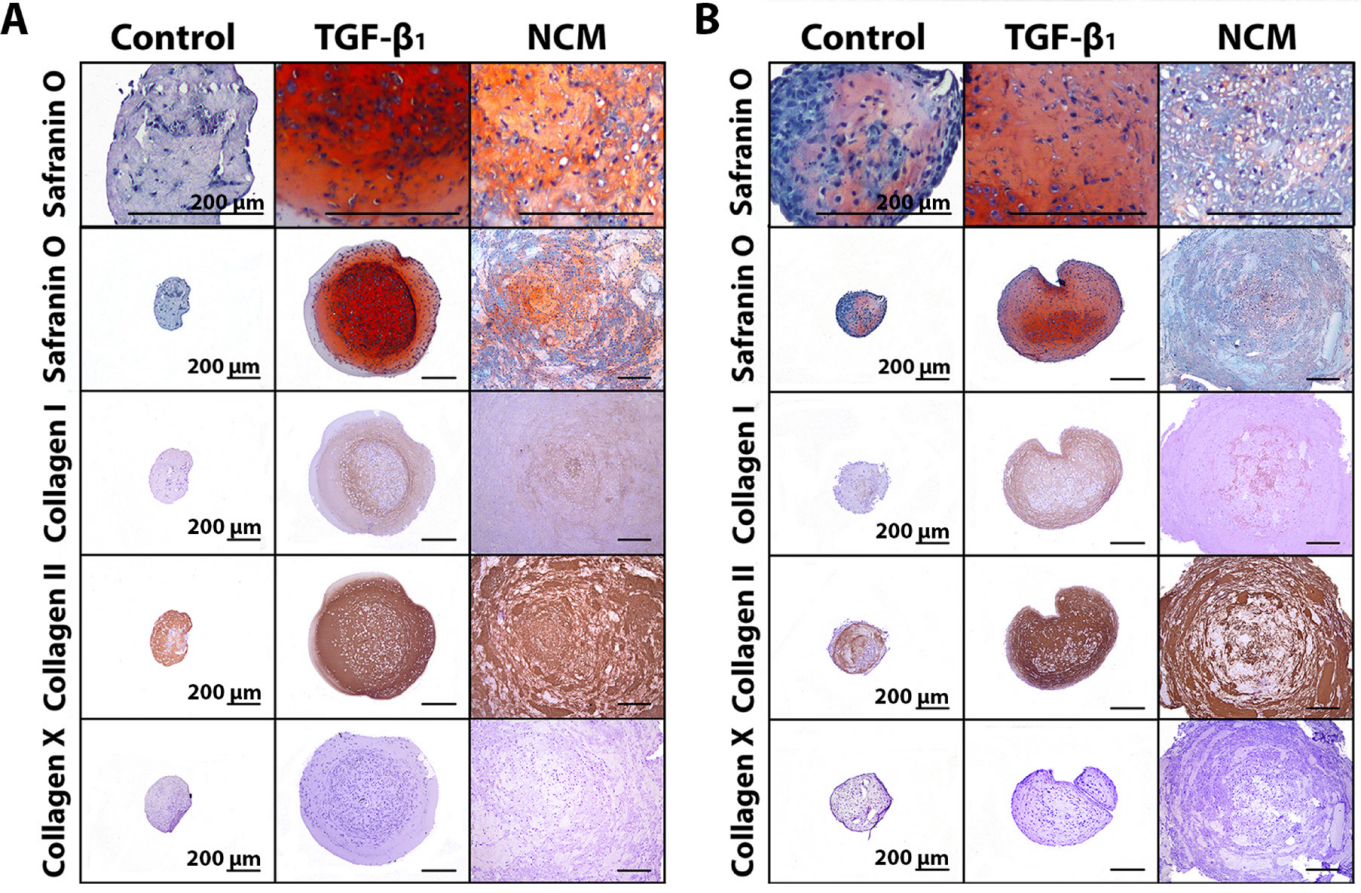
Notochordal cell-derived matrix (NCM) induces extracellular matrix deposition by chondrocyte-like cells (CLCs) Safranin O/Fast Green staining and collagen type I, II, and X immunohistochemistry of canine (**A**) and human (**B**) CLC micro-aggregates of 35,000 cells cultured in basal culture medium (negative control), supplemented with 10 ng/mL TGF-β_1_ (positive control), and 10 mg/mL NCM for 28 days. *n* = 6 per species, in duplicates. All scale bars indicate 200 µm.

In the current study, 10 ng/mL TGF-β_1_ was used as positive control to demonstrate the chondrogenic capacity of the donors employed, and indeed increased the DNA and GAG content of the micro-aggregates and induced proteoglycan synthesis in human and canine CLCs (*p* < 0.05, Figure 1A–1H). NCM increased the DNA, GAG, and GAG/DNA content of canine and human CLC micro-aggregates after 28 days of culture (*p* < 0.05, Figures 1A–1F and 2); the cells demonstrated a typical chondrocyte-like appearance. Given that NCM is rich in ECM components, we explored whether CLCs produced and/or incorporated the matrix provided during culture. The ^35^SO_4_^2-^ incorporation assay demonstrated that NCM-treated human CLCs synthesized less proteoglycans than controls (*p* < 0.001; Figure 1H), which may indicate that GAGs were incorporated in the micro-aggregates from NCM, instead of being synthesized by the human CLCs. In contrast, NCM induced proteoglycan synthesis in canine CLCs (*p* < 0.01, Figure 1G), indicating that they (also) synthesized GAGs themselves. Collagen type I and II deposition appeared increased in 28-day NCM-treated CLC micro-aggregates whereas collagen type X (hypertrophy marker) was not observed (Figure 2). Altogether, NCM induced GAG and collagen type II-rich matrix deposition in CLCs *in vitro*.

### NCM supports MSC chondrogenesis *in vitro*

**Figure 3:**
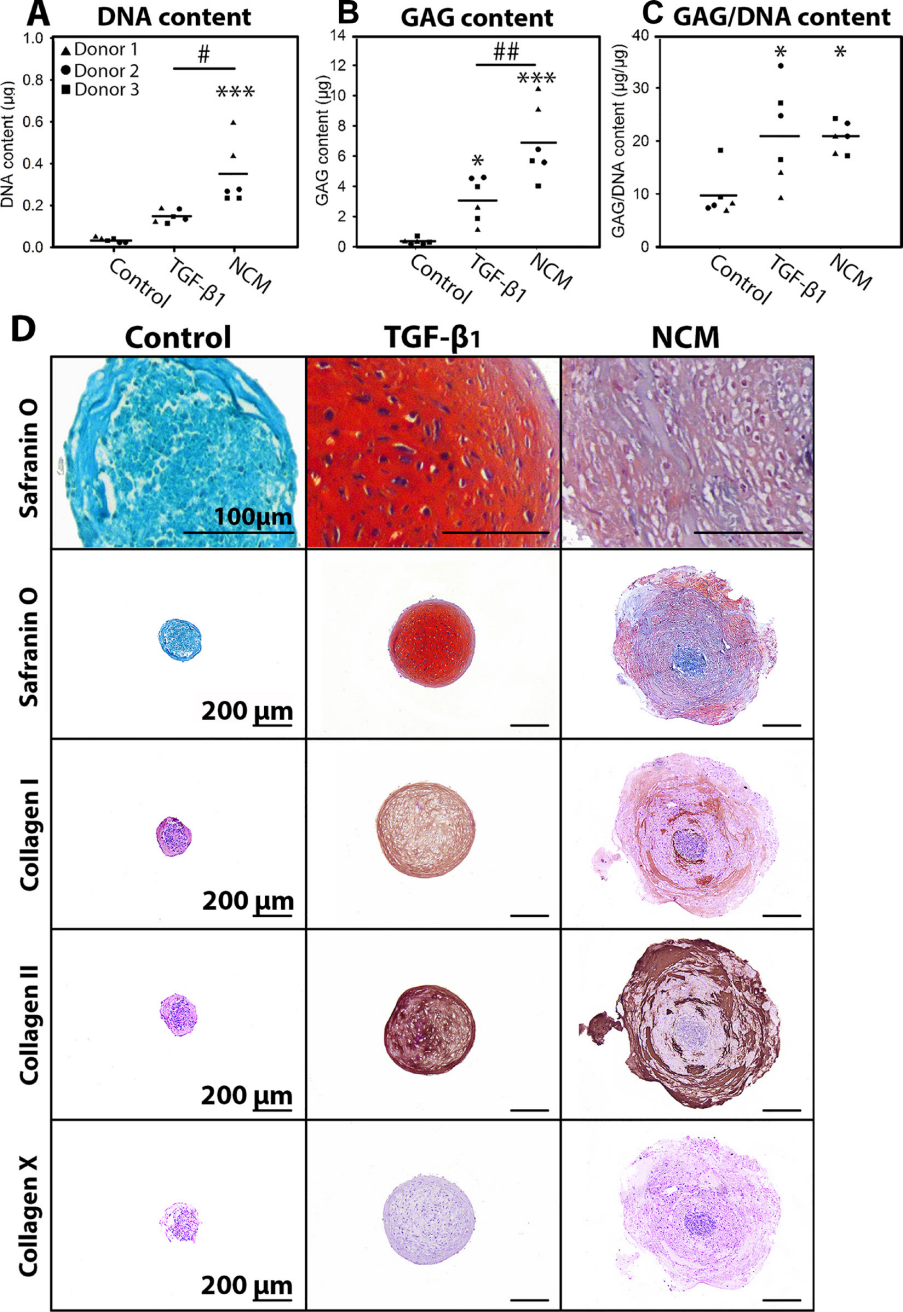
Notochordal cell-derived matrix (NCM) facilitates chondrogenic differentiation of canine MSCs DNA (**A**), glycosaminoglycan (GAG; **B**) and GAG/DNA (**C**) content. MSC micro-aggregates of 35,000 cells were cultured in (negative) control culture medium, supplemented with 10 ng/mL TGF-β_1_ (positive control) or 10 mg/mL NCM for 28 days. (**D**) Safranin O/Fast Green staining and collagen type I, II, and X immunohistochemistry at day 28. ^*,***^p < 0.05, *p* < 0.001, respectively, for the indicated condition versus controls; ^#,##^*p* < 0.05 and *p* < 0.01, respectively, for the two indicated conditions by the horizontal bar; *n* = 3 in duplicates; mean values are given. ANOVA (normally distributed data), Kruskal Wallis/Mann–Whitney U (non-parametric data), and Benjamini & Hochberg tests were performed.

The effect of NCM was tested on MSCs from three canine donors. Positive control treatment (TGF-β_1_) increased the GAG and GAG/DNA content of MSC micro-aggregates versus controls (*p* < 0.05, Figure 3B–3D), indicating successful chondrogenic differentiation. The DNA and GAG content of NCM-treated micro-aggregates was higher versus TGF-β_1_-treated micro-aggregates (*p* < 0.05, Figure 3A–3B). The GAG/DNA content of micro-aggregates treated with TGF-β_1_ or NCM was increased versus controls (*p* < 0.05), with no differences between conditions (Figure 3C). Additionally, both TGF-β_1_ and NCM induced collagen type I and II deposition, whereas collagen type X was not deposited (Figure 3D). Lastly, NCM-treated micro-aggregates contained phenotypically chondrocyte-like cells, surrounded by collagen type II and GAG/rich extracellular matrix (Figure 3D). Altogether, NCM supported chondrogenesis of canine MSCs *in vitro*.

### MSC characterization

To demonstrate that bone marrow-derived stromal cells from the male Beagle donor injected in the female Beagle IVDs met requirements to be defined as MSCs, multilineage differentiation and FACS analysis was performed [19]. Chondrogenic, osteogenic, and adipogenic differentiation were successfully induced, and cells were CD34^−^, CD45^−^, CD29^+^ and CD90^+^ (Supplementary File 1). Only 4.4% of cells were CD105^+^, possibly explained by low cross-species reactivity of anti-human CD105 used due to unavailability of commercial anti-canine CD105 [20]. Altogether, the results confirmed the presence of MSCs in the intradiscally injected cells.

### Induction of IVD degeneration *in vivo*

To enable studying the effect of the treatments on mildly, and also on more severely degenerated IVDs, six weeks before the start of the experiment (T = −1.5 months), moderate IVD degeneration was induced by partial NP removal (NX) in five IVDs per Beagle dog (NX-IVDs; *n* = 6 dogs, Supplementary File 2A–2B). Six weeks later (T = 0 months), the noNX-IVDs (IVDs in which no NX was performed) and the NX-IVDs were either not injected (controls) or injected with NCM, MSCs, or NCM+MSCs (Supplementary File 2G–2H). At T = 3 months, two NCM-treated IVDs per dog received an additional NCM injection. One Beagle died unexpectedly at T = 0 (cause of death unrelated to treatment) and the IVDs of this dog were used as baseline values for moderate (induced) IVD degeneration (*n* = 1 dog). At T = 0 months, the AF appeared not affected by the induction of moderate IVD degeneration (Supplementary File 2D, 2I–2M). In contrast, NPs of NX-IVDs showed macroscopically a brown discoloration (Supplementary File 2D), histologically less intense Alcian blue (GAG) and more intense Picrosirius Red (collagen) staining (Supplementary File 2E–2F), and biochemically a decreased GAG and GAG/DNA content (*p* ≤ 0.05; Supplementary File 2I, 2K) in comparison with NPs of noNX-IVDs, indicating that degeneration was successfully modified from mild (spontaneous) to moderate (induced) IVD degeneration. Lastly, it was confirmed in two IVDs (Th12-13, NX and Th13-L1, noNX) of the female dog, which died immediately post-operatively due to unknown reasons, that injected male MSCs could be detected (SRY DNA), indicating proper intradiscal MSC injection and tracking (Supplementary File 2N).

### The effect of NCM on degenerated canine IVDs *in vivo*

**Figure 4:**
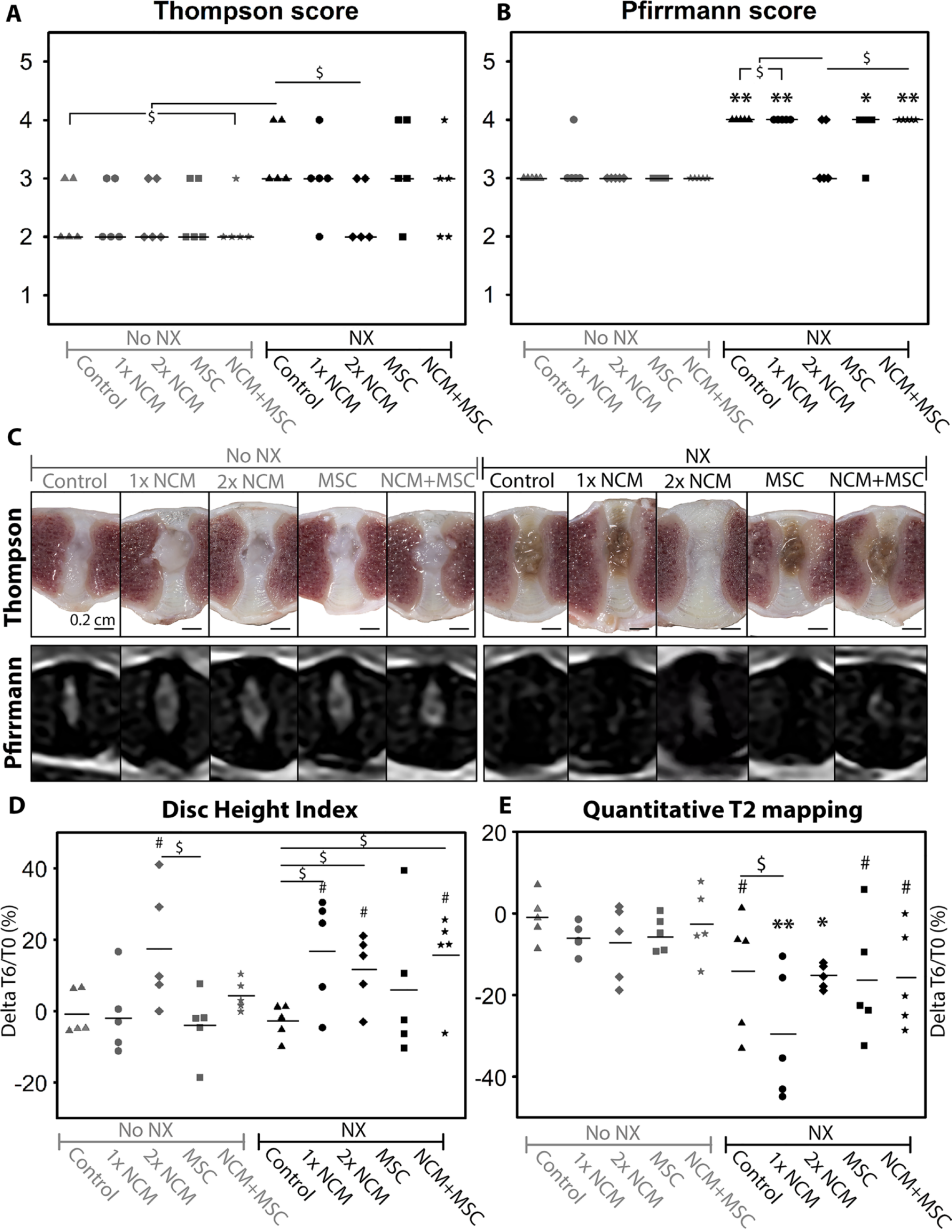
Notochordal cell-derived matrix (NCM) improves the macroscopic Thompson score and radiologic parameters of degenerated intervertebral discs (IVDs) Median (Thompson (**A**), Pfirrmann (**B**)) and mean (Disc Height Index; DHI (**D**), T2 values (**E**)) values are indicated. (**C**) Macroscopic and MRI pictures of the canine IVDs. Change in DHI and T2 mapping values were calculated at individual intervertebral disc (IVD) level. NX: partial nucleus pulposus (NP) removal to induce moderate IVD degeneration. ^*,**^p < 0.05, *p* < 0.01, respectively for the indicated condition versus control noNX-IVDs; ^#^*p* = 0.05–0.15, large effect size, for the indicated condition versus control noNX-IVDs; ^$^*p* = 0.05–0.15, large effect size, between indicated conditions; *n* = 5. For Thompson and Pfirrmann data, a Mann–Whitney Wilcoxon test and for DHI and quantitative T2 mapping, a Cox proportional hazard model (donor as random effect) was performed with Benjamini & Hochberg tests. For comparisons with 0.05 ≥ *p* ≤ 0.20, effect sizes (Cliff’s delta) were determined.

At T = 6 months, the effect of the different treatments (control, 1xNCM, NCM reinjection after three months (2xNCM), MSC, NCM+MSC) on mildly and moderately degenerated canine IVDs was determined using macroscopic, radiologic, histologic and biochemical analysis (*n* = 5 dogs). First, the IVDs were scored according to macroscopic Thompson (grade 1–5) [21] and MRI-based Pfirrmann (grade 1–5) [22] grading validated for dogs; grades increase with degeneration. At T = 6 months, control NX-IVDs had a higher median Thompson and Pfirrmann score than control noNX-IVDs (*p* = 0.08 with large effect size (ES) and *p* < 0.01, respectively; Figure 4A–4B), indicating that more severe IVD degeneration was induced with NX. The Thompson and Pfirrmann score of noNX-IVDs did not improve by any treatment. In contrast, 2xNCM-treated NX-IVDs tended to show a lower median Thompson and Pfirrmann score than control NX-IVDs (*p* = 0.08 and 0.15, large ES) and did not differ from control noNX-IVDs indicating reversal or halting of the degenerative process. In line with this, all NX-IVD NPs showed a brown discoloration (compatible with IVD degeneration), except 2xNCM-treated IVDs in four out of five dogs (Figure 4C). Altogether, 2xNCM improved the Thompson and Pfirrmann score of moderately degenerated IVDs.

The Disc Height Index (DHI) did not significantly change from T = 0 until T = 6 months in control-, 1xNCM-, MSC- and NCM+MSC-treated noNX-IVDs, but tended to increase in time by 2xNCM (*p* = 0.14, large ES; Figure 4D). In control NX-IVDs, the DHI did not considerably change from T = 0 to T = 6 months. In 1xNCM-, 2xNCM- and NCM+MSC-treated, but not MSC-treated NX-IVDs, the increase in DHI tended to be higher versus control NX- and noNX-IVDs (*p* = 0.06–0.09, large ES), indicating a NCM specific effect.

T1ρ and T2 MRI values were employed for quantitative MRI analysis. T1ρ values did not significantly change in time, regardless the condition (mean: 0.59, SD: 0.14). T2 values were not different between T = 0 and T = 6 months in noNX-IVDs (Figure 4E). In all NX-IVDs, T2 values decreased in time versus control noNX-IVDs (*p* < 0.05, or *p* = 0.05–0.08 with large ES). Altogether, induction of degeneration reduced T2 values, but no treatment was able to increase T2 values during the six months follow up period.

Lastly, the presence and progression of Modic Changes (MCs), periosteal bone formation, ventral spondylosis and EP lysis was recorded (Supplementary File 3). In noNX-IVDs, MCs or EP lysis were not induced by any of the treatments. Periosteal bone formation and mild ventral spondylosis was observed only in three 1xNCM-treated noNX-IVDs and two NCM+MSC-treated noNX-IVDs. As expected, the NX procedure induced all parameters, while 2xNCM did not induce any of the parameters in NX-IVDs.

**Figure 5:**
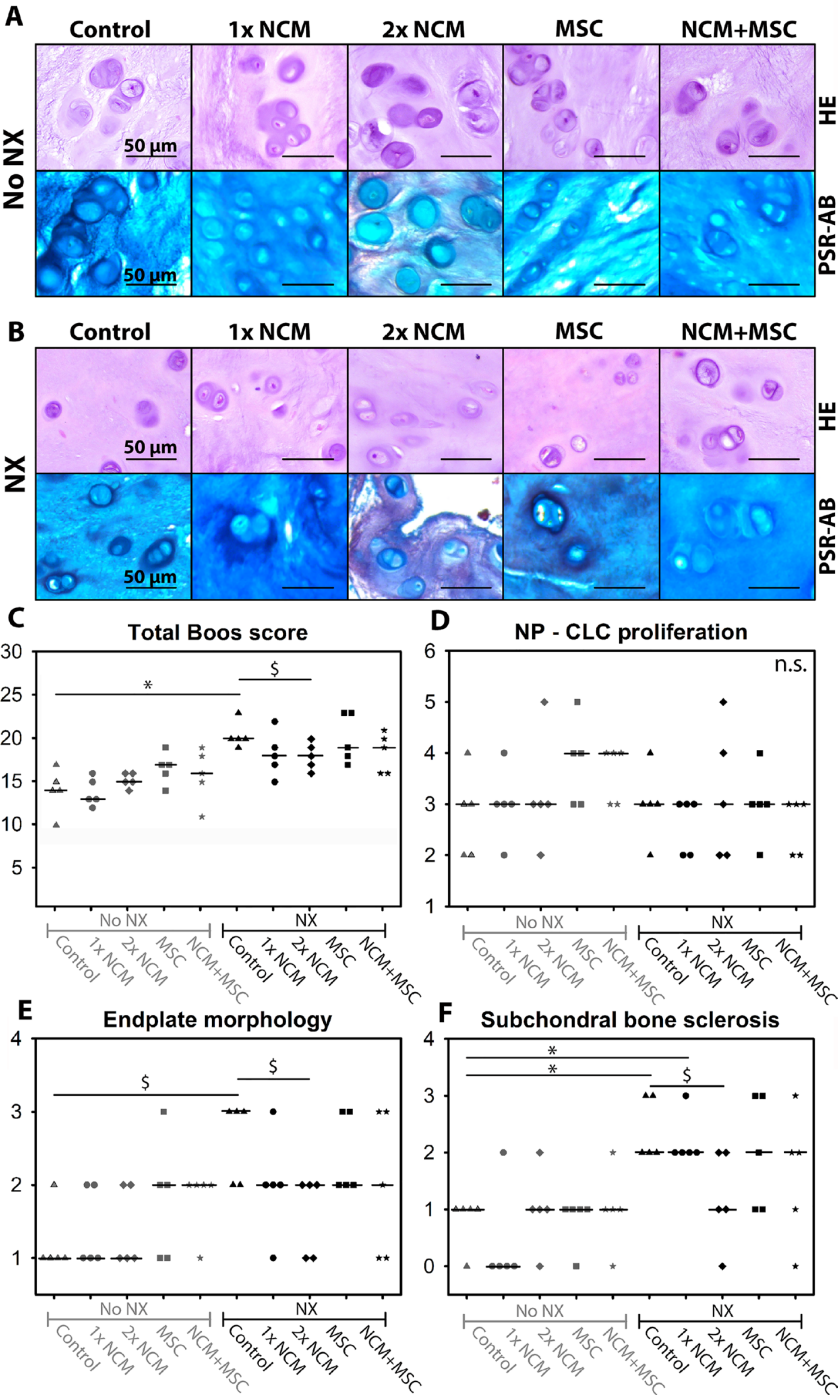
Notochordal cell-derived matrix (NCM) improves canine intervertebral disc (IVD) histology H&E and Picrosirius Red-Alcian Blue (blue: glycosaminoglycan, red: collagen) staining of canine nucleus pulposus tissue (**A**, **B**). Total histologic Boos score (**C**) and its subsets: chondrocyte-like (CLC) proliferation in the nucleus pulposus (NP; **D**), end plate morphology (**E**), and subchondral bone sclerosis (**F**). Median values are indicated. NX: partial NP removal to induce moderate IVD degeneration. n.s.: not significant; ^*^p < 0.05 between indicated conditions; ^$^*p* = 0.05–0.18 with medium-large effect size between indicated conditions; *n* = 5. Data were analyzed with Mann–Whitney Wilcoxon and Benjamini & Hochberg tests. For comparisons with 0.05 ≥ *p* ≤ 0.20, effect sizes (Cliff’s delta) were determined.

For histological analysis, all IVDs were scored (Boos grading validated for dogs [23]; score increases with degeneration (0–29)). At T = 6 months, only CLCs were present in all NPs (Figure 5A, 5B). Only in 2x NCM-treated NPs, Picrosirius Red (collagen) staining was observed (Figure 5A, 5B). Furthermore, the median total Boos score significantly increased in control NX-IVDs versus control noNX-IVDs (*p* < 0.05; Figure 5C) with six points (from 14 to 20), indicating that moderate IVD degeneration was induced. Only 2xNCM tended to improve the median Boos score compared with control NX-IVDs with three points (*p* = 0.18, medium ES). Boos score subcriteria indicated no differences between conditions for AF morphology, CLC morphology and proliferation in the NP, presence of NCs (none), matrix staining of the NP, and new bone formation (Figure 5A, 5B, 5D). The histologic score for chondrocyte metaplasia and tear and cleft formation in the AF (data not shown) and EP morphology (irregularity, thickness; Figure 5E) of control NX-IVDs tended to be increased versus control noNX-IVDs (*p* = 0.12–0.16, medium-large ES). Additionally, the median subchondral bone sclerosis score was significantly increased in control NX-IVDs versus control noNX-IVDs (*p* < 0.05; Figure 5F). In NX-IVDs, 2xNCM tended to improve the median EP morphology and subchondral bone sclerosis score versus controls (*p* = 0.18, medium ES). Taken together, EP irregularity and bone sclerosis was induced by NX, while 2xNCM inhibited these pathological processes.

**Figure 6:**
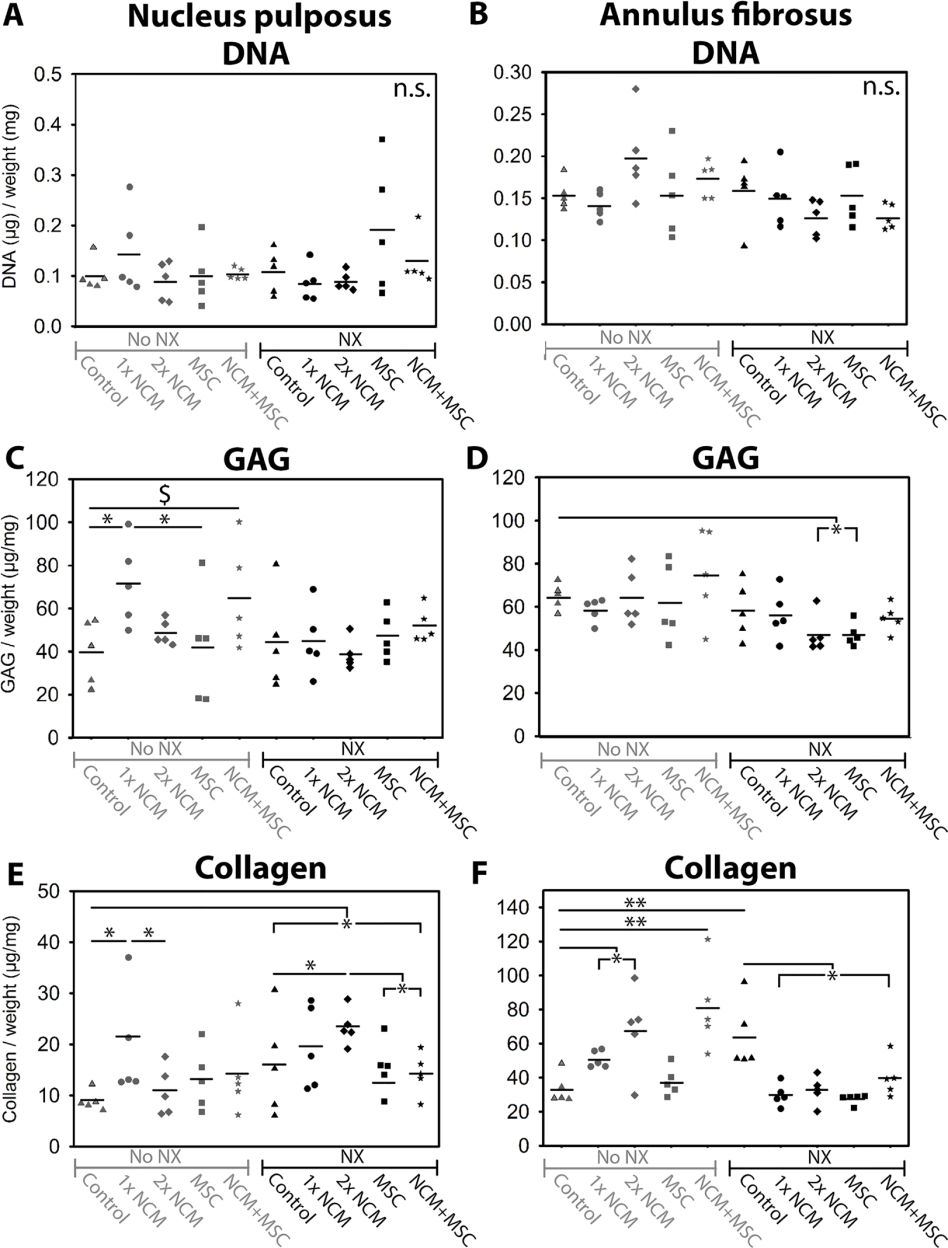
The effect of notochordal cell-derived matrix (NCM) on the DNA (**A**, **B**), glycosaminoglycan (GAG; **C**, **D**), and total collagen (**E**, **F**) content of the nucleus pulposus (NP) and annulus fibrosus (AF). Mean values are indicated. NX: partial NP removal to induce moderate IVD degeneration. n.s.: not significant; ^*,**^p < 0.05, *p* < 0.01, respectively, between indicated conditions; ^$^*p* = 0.07, large effect size between indicated conditions; *n* = 5. Data were analyzed with randomized block design ANOVAs and Benjamini & Hochberg tests. For comparisons with 0.05 ≥ *p* ≤ 0.20, effect sizes (Hedge’s g) were determined.

At T = 6 months, the DNA content of the NP and AF tissue did not significantly differ, regardless the treatment and induction of degeneration (Figure 6A, 6B). SRY DNA was undetectable in the female IVDs in which male MSCs were injected, indicating that the male MSCs had not survived.

The NP and AF GAG content was not different between noNX-IVDs and NX-IVDs at T = 6 months (Figure 6C–6D), but was significantly lower in NPs from NX-IVDs versus NPs from noNX-IVDs (*p* < 0.05, Supplementary File 2I, 2K) at T = 0 months (six weeks after NX). Thus, the decreased GAG content of NPs from IVDs in which moderate degeneration was induced (observed at T = 0 months) recovered to baseline levels at T = 6 months, suggesting an attempt at repair. This repair was further augmented by NCM: at T = 6 months, 1xNCM significantly increased the NP GAG content in noNX-IVDs versus control- and MSC-treated noNX-IVDs (*p* < 0.05). Also NCM+MSC treatment tended to increase the NP GAG content versus control noNX-IVDs (*p* = 0.07 with large ES). Moreover, in NX-IVDs, MSC and 2xNCM treatment significantly decreased the AF GAG content compared with noNX-control IVDs (*p* < 0.05), indicating less AF chondrification, a hallmark of the degenerative process.

**Figure 7:**
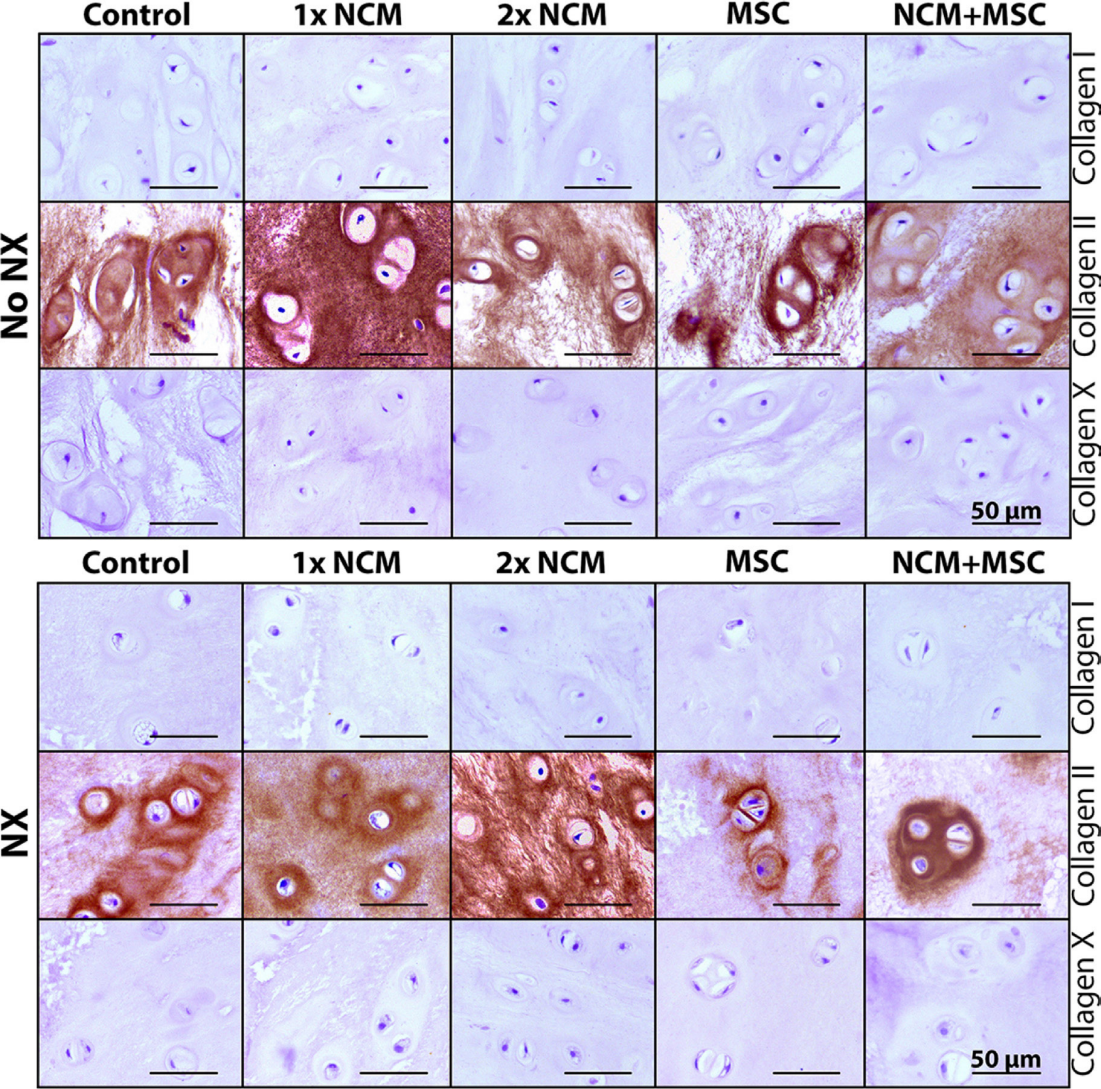
Notochordal cell-derived matrix (NCM) stimulates collagen type II, but no collagen type I or X deposition in the canine nucleus pulposus (NP) *in vivo* NX: partial NP removal to induce moderate IVD degeneration. *n* = 5.

The collagen content of control NX-IVDs was significantly higher versus control noNX-IVDs (NP: *p* < 0.05, AF: *p* < 0.01), indicating that collagen deposition increased with degeneration (Figure 6E–6F). In noNX-IVDs, 1xNCM significantly increased the NP collagen content versus control and 2xNCM (*p* < 0.05). Additionally, 1xNCM, 2xNCM, and NCM+MSC significantly increased the AF collagen content versus controls in noNX-IVDs. In NX-IVDs, 2xNCM significantly increased the NP collagen content versus control, MSC, and MSC+NCM (*p* < 0.05). Lastly, the AF collagen content was significantly reduced by all treatments versus controls in NX-IVDs (*p* < 0.05). To determine the types of collagen that were deposited, immunohistochemistry was performed. Collagen type I was present in the AF (not differentially expressed; data not shown), but not in the NP (Figure 7), suggesting the absence of NP fibrosis. Collagen type II was present in all NPs (Figure 7). In noNX-IVD NPs, collagen type II was most abundantly present after 1xNCM treatment, while in NX-IVD NPs it was most abundantly deposited by 1xNCM and 2xNCM. Collagen type X was undetectable regardless the treatment (Figure 7), indicating absent hypertrophic differentiation.

Altogether, NCM induced collagen type II-rich matrix deposition *in vivo*. NCM is enriched in collagen and GAGs. Injection of 50 µL of 10 mg/mL NCM (containing both 5 mg/mL GAGs and collagen) would contribute to 0.25 mg GAG and collagen per treated IVD. While 2xNCM increased the NP collagen content, it did not augment the NP GAG content of NX-IVDs. Since GAGs attract water and the biochemical data were corrected for wet weight, we possibly missed the presence of an increased GAG content (per mg dry weight).

At T = 6 months, no differences in NP mRNA expression were established between conditions for ECM-related genes *SOX9, ACAN, COL1A1, COLX, ADAMTS5* and *MMP13* (data not shown). This indicates the limited translatability of gene expression data in view of the long-term follow-up period of six months. *COL2A1* mRNA expression was, however, increased in all NX-IVDs versus control noNX-IVDs (*p* < 0.05 or *p* = 0.07 with large ES; Supplementary File 4A). In NX-IVDs, 2xNCM tended to increase *COL2A1* expression versus all other treatments (0.05 ≥ *p* ≤ 0.17, medium ES). *TIMP* expression tended to be increased in NX-IVDs treated with 1xNCM, 2xNCM, MSCs and NCM+MSCs versus control noNX-IVDs (*p* < 0.05, or *p* = 0.14 with medium ES; Supplementary File 4B). Altogether, NCM induced an anabolic effect by increasing the expression of the anti-catabolic *TIMP* gene and by inducing *COL2A1* expression. The latter is in line with the augmented collagen type II deposition (IHC). Expression of the cell proliferation- and apoptosis-related genes *CCND1*, *CASP3, BCL2,* and *BAX* was not different between conditions (data not shown). Regarding NP specific markers, 2xNCM induced *KRT18* and *KRT19* expression in mildly degenerated IVDs, and reduced their expression in moderately degenerated IVDs (*p* = 0.01–0.10, medium-large ES; Supplementary File 4C–4D). *PAI1* expression was significantly induced by 2xNCM in NX-IVDs (*p* < 0.05; S4E), suggesting increased TGF-β signaling at mRNA level.

**Figure 8:**
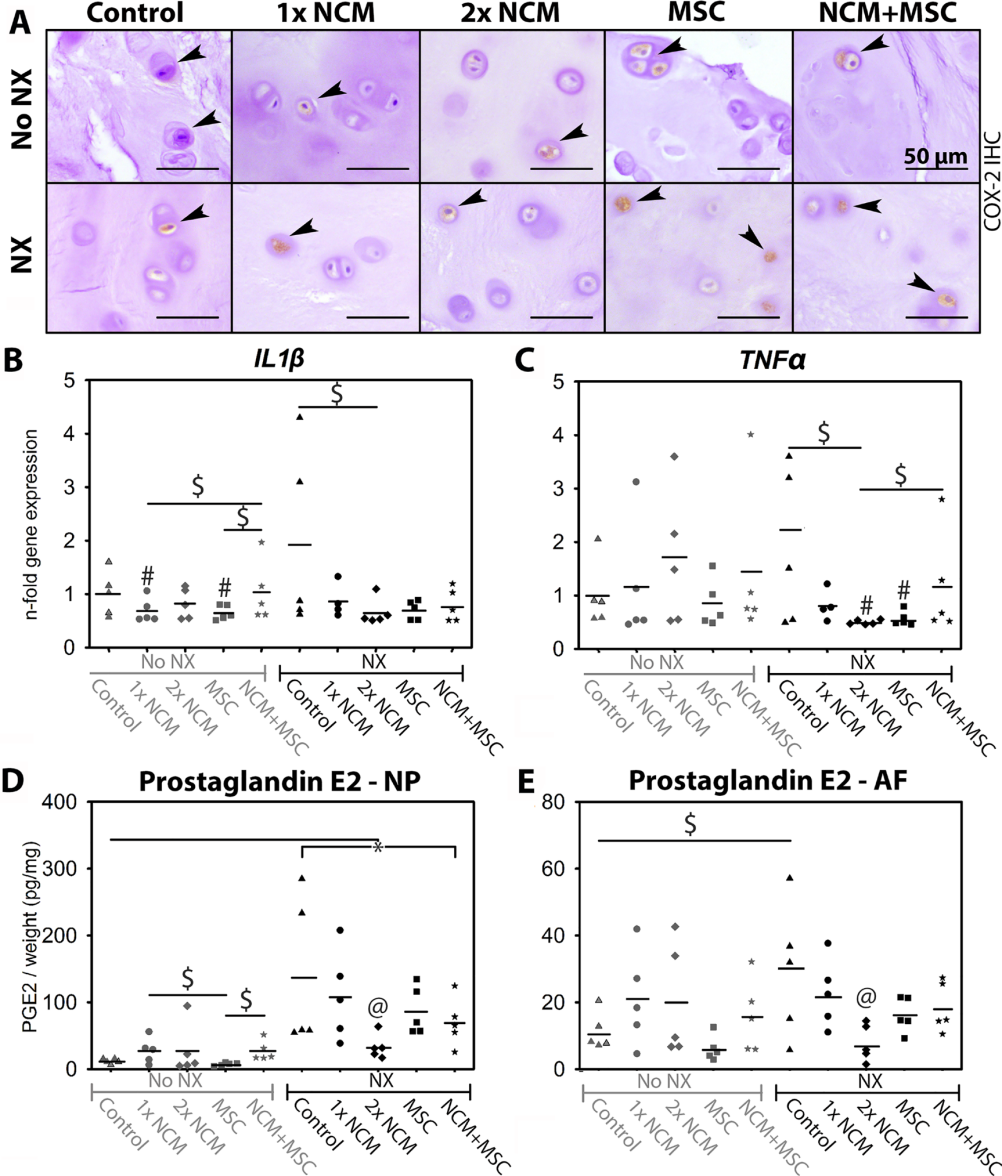
Notochordal cell-derived matrix (NCM) ameliorates inflammation in canine intervertebral discs (IVDs) *in vivo* Cyclo-oxygenase-2 (COX-2) positive chondrocyte-like cells (CLCs) in the canine nucleus pulposus (NP) are indicated with arrowheads (**A**). Relative *IL1β* (**B**) and *TNFα* (**C**) expression (control noNX-IVDs were set at 1) of canine NPs, and prostaglandin E2 (PGE2) content (**D**, **E**) of canine IVDs at T = 6 months are given with indicated mean values. AF: Annulus fibrosus. NX: partial NP removal to induce moderate IVD degeneration. ^*^p < 0.05 between indicated conditions; ^#^different from control noNX-IVDs (0.05 ≥ *p* ≤ 0.20, medium/large ES); $: difference between indicated conditions with medium/large effect size (0.05 ≥ *p* ≤ 0.20); @: different from all other conditions in which NX was performed (*p* < 0.05 or *p* < 0.1 with large effect size), *n* = 5. PGE2 data were analyzed with a randomized block design ANOVA. For RT-qPCR data, a Cox proportional hazard test was performed (donor random effect). For comparisons with 0.05 ≥ *p* ≤ 0.20, effect sizes (Hedge’s g and Cliff’s delta) were determined. Benjamini & Hochberg tests were performed.

To determine the treatment effect on inflammation, cycloxygenase-2 (COX-2) IHC and RT-qPCR were performed, and the prostaglandin E2 (PGE2) content of the IVD tissues was determined. COX-2 immunopositivity was present in every NP (Figure 8A) and AF. On average, 7–20% of canine CLCs in the NP expressed cytoplasmic COX-2, with no differences between conditions (data not shown). *IL1β* and *TNFα* (pro-inflammatory cytokines) expression was not different between control noNX- and NX-IVD NPs (Figure 8B–8C). In NX-IVDs, however, 2xNCM tended to reduce *IL1β* and *TNFα* expression (*p* = 0.09 and 0.11, medium ES). Control NX-IVDs showed a higher PGE2 content than control noNX-IVDs (NP: *p* < 0.05, AF: *p* = 0.06, very large ES; Figure 8D–8E), indicating that PGE2 levels (indicative of increased COX-2 activity) increased with degeneration. In line with RT-qPCR results, 2xNCM significantly decreased the NP and AF PGE2 content in NX-IVDs (*p* < 0.05, large effect size). Altogether, NCM ameliorated inflammation in moderately degenerated IVDs.

## DISCUSSION

The spine field has already demonstrated the regenerative potential of NCs [5–12]. The present innovative study demonstrates that by employing healthy NC-derived NP tissue matrix, we can harness the NC regenerative potential and exploit it for biological IVD repair. As a first step towards bench-to-bedside translation, we demonstrate that NCM exerted regenerative effects on canine and human CLCs from degenerated IVDs *in vitro* and on canine IVDs *in vivo* by inducing anabolic effects and ameliorating inflammation. The question arises how NCM exerts its anabolic effects. It may serve as ‘instructive matrix’, locally increasing growth factor concentrations and promoting their biological activity [24]. In this respect, NCM may be comparable to DMB, which is successfully employed in clinical practice. DBM induces bone regeneration by providing a degradable matrix that facilitates the release and modulates the accessibility of growth factors normally present in bone [15]. Increased *PAI1* (TGF-β pathway target gene) expression in 2xNCM-treated IVDs indeed indicates augmented Smad signalling. The exact mechanism of action of NCM remains to be determined and is most probably a combination of bioactive factors retained within NCM and ECM molecules modulating their activity [25].

### MSCs do not exert distinct effects in the degenerated canine IVD *in vivo*

Intradiscal allogeneic MSC delivery is considered a promising IVD regeneration strategy. MSC transplantation was not accompanied by osteophyte formation (a possible complication [26]), most probably because a polymerized hydrogel, which prevents leakage, was used as carrier or because the MSCs did not thrive *in vivo*. Although we detected male DNA (MSCs) in the female IVDs immediately after intradiscal injection, male DNA was undetectable after six months. This result contrasts with previous Beagle studies, in which autologous transplanted MSCs could be traced back up to twelve months and exerted beneficial effects [27–29]. This contradiction, however, needs to be cautiously interpreted, since only one quart of IVD tissue was available for SRY analysis, so low numbers of surviving MSCs were possibly missed. Nevertheless, transplanted MSCs, capable of multilineage differentiation *in vitro*, did not exert beneficial effects *in vivo*, alone or combined with NCM. MSC populations are known to exhibit considerable donor-to-donor and intra-population heterogeneity [30], and in the harsh environment of the degenerated IVD, this could have contributed at best to a transient trophic effect which was not biologically relevant at longterm follow-up.

### Intradiscally applied NCM has a regenerative effect on degenerated canine IVDs

The present study demonstrates that NCM exerted regenerative effects on MSCs and CLCs derived from degenerated IVDs *in vitro*. Although canine CLCs seemed to be more responsive to NCM than human CLCs, NCM exerted an anabolic effect and increased the DNA content (number of cells/micro-aggregate) in both species, in line with previous work on decellularized bovine NP ECM [31].

Based on these promising results, an *in vivo* study was performed. In mildly degenerated IVDs, NCM upregulated mRNA expression of NP-specific markers, augmented the NP GAG and collagen content, and hence seemed to favor IVD health. Notably, in moderately degenerated NX-IVDs, NCM-mediated effects were more pronounced. Mainly 2xNCM exerted beneficial effects on the NPs from NX-IVDs: the macroscopic Thompson, histologic Boos (total, end plate morphology, and subchondral bone sclerosis score) and MRI-based Pfirrmann score were improved. Additionally, the DHI of 1xNCM-, 2xNCM- and NCM+MSC-treated NX-IVDs increased during the study. Given that MSC injection alone was not able to increase the DHI, this beneficial effect is presumably NCM-specific. Since 2xNCM induced collagen type II, but no collagen type I and X expression, NP-like ECM, but no fibrotic or hypertrophic ECM [32, 33] was deposited. Lastly, 2xNCM was able to exert protective effects on the L7-S1 IVD, a junction that permits the highest range of motion [34] and predisposes this IVD to a high degree of wear and tear. Altogether, these findings imply an NCM-dependent regenerative response, resulting in beneficial quantitative and qualitative ECM changes.

### Quantitative T1ρ and T2 mapping did not detect NCM-mediated effects

In contrast with other readout parameters, T1ρ and T2 mapping did not detect NCM-mediated regenerative effects. T1ρ is sensitive for interactions between macromolecules (*e.g.* GAGs) and water [35], while T2 mapping provides information regarding collagen, water and GAG content, ECM structure and orientation [36]. With the induction of degeneration, advanced glycation end products (AGEs) presumably accumulated [37, 38] and the NP collagen content increased, whereas T2 values decreased. These results are in agreement with previous studies demonstrating a negative correlation between T2 values and AGE accumulation [39]/collagen content [40, 41]. Thus, IVD degeneration was detected by quantitative T2 mapping. In contrast, NCM-mediated regeneration (observed macroscopically, histologically, biochemically and according to MRI-based Pfirrmann grading) was not detected with T1ρ or T2 mapping. Noteworthy, T1ρ and T2 mapping have been specifically validated for IVD degeneration, but not for regeneration, which does not necessarily follow an identical reverse process. Lastly, T1ρ and T2 mapping are influenced by ECM structure/orientation and the water, GAG (both positively correlated with T2 vaules), and collagen (negatively correlated with T2 values) content of the NP [35, 36], making it difficult to detect regenerative changes when more than one of these parameters is affected by the treatment.

### NCM exerts anti-inflammatory effects in degenerated canine IVDs *in vivo*

Besides beneficial effects on macroscopic, radiologic, biochemical, and histological level, NCM also exerted anti-inflammatory effects on degenerated IVDs *in vivo*. The PGE2-producing enzyme COX-2 was uniformly present in NPs, implying local PGE2 synthesis. PGE2 levels were, however, decreased by 2xNCM in NX-IVD NPs, indicating that 2xNCM decreased COX-2 activity and inhibited inflammation. In line with this, all NX-IVD NPs, except 2xNCM-treated NPs, showed a brown discoloration. This brown color was presumably caused by accumulation of AGEs. AGEs are formed through non-enzymatic glycation of amino residues and oxidation of fatty acids during IVD degeneration [37, 38] and can act as pro-inflammatory mediator [42]. Altogether, 2xNCM may prevent AGE accumulation in degenerated IVDs, thereby exerting an anti-inflammatory effect. This hypothesis is confirmed by the decreased PGE2 content and downregulated IL1β and TNFα expression in 2xNCM-treated NX-IVDs. These anti-inflammatory NC properties have already been demonstrated previously [43].

### Limitations and future directions

The present study demonstrates that 10 mg/mL NCM exerted beneficial effects on canine and human CLCs from degenerated IVDs *in vitro* and degenerated Beagle IVDs *in vivo*. Given that 0.5 mg NCM/IVD exerted beneficial effects, it is tempting to hypothesize that higher NCM dosages may further improve the regenerative and anti-inflammatory effects. After fine-tuning the dose-dependent efficacy of NCM, follow-up studies should look into the application of NCM in hydrogel form, which reduces the risk of leakage after intradiscal injection. Furthermore, for safe (veterinary and human) clinical application, removal of nucleic acid from NCM should be achieved with preservation of bioactivity [31].

## MATERIALS AND METHODS

### Overall study design

**Figure 9:**
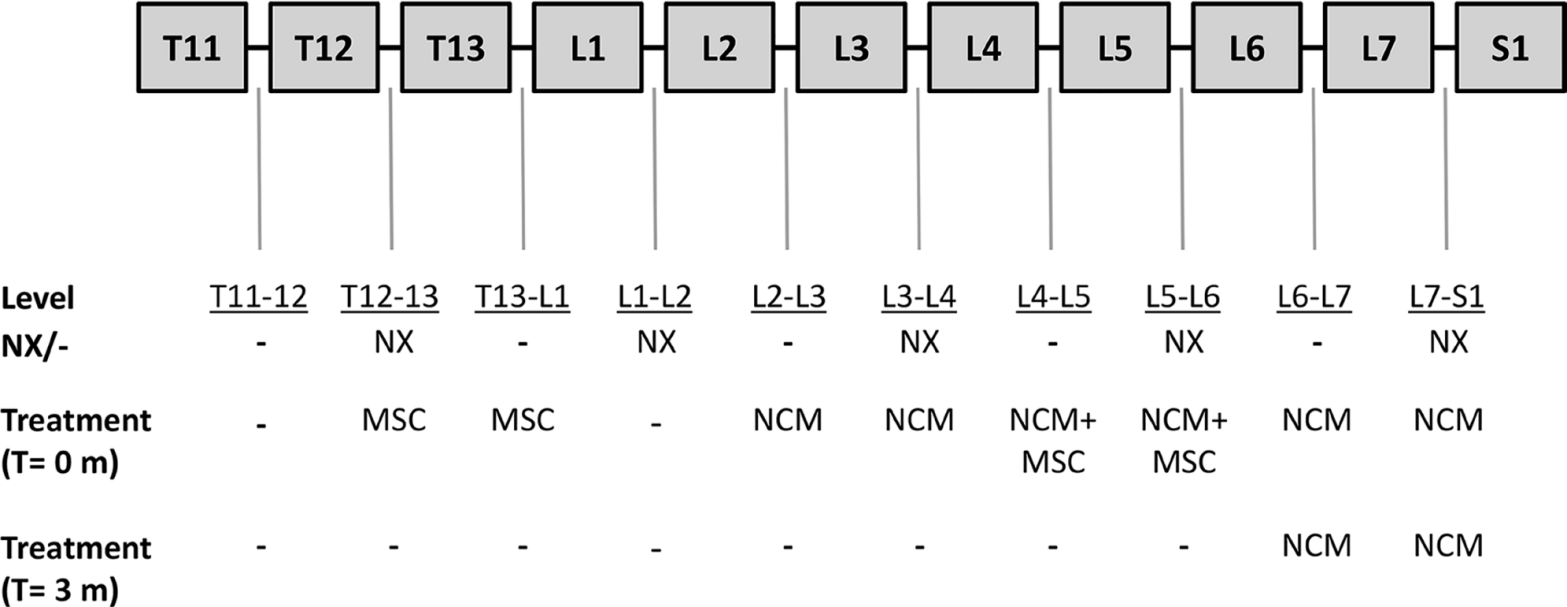
Overview of the intradiscal treatment per canine intervertebal disc (IVD) Six weeks before the start of the experiment (T = −1.5 months), more severe (moderate) IVD degeneration was induced in five IVDs per dog by partial nucleus pulposus (NP) removal (NX; T12-T13, L1-L2, L3-L4, L5-L6, and L7-S1) on the left side of the spine. At the start of the experiment (T = 0 months), the mildly and moderately degenerated canine IVDs were injected on the right side of the spine with 50 µL of (a) 1^*^10^6^ canine mesenchymal stromal cells (MSCs) incorporated in an albumin-based hydrogel, (b) 10 mg/mL notochordal cell-derived matrix (NCM), or (c) 1^*^10^6^ canine MSCs suspended in 10 mg/mL NCM. Three months after the first injections (T = 3 months), two IVDs per dog (L6-L7 and L7-S1) were again injected with 50 µL of 10 mg/mL NCM. Every dog received a similar treatment per spinal location. -: no NX/no treatment. *n* = 6 dogs. One dog died unexpectedly at T = 0 (cause of death unrelated to treatment) and the IVDs of this dog were used as baseline values. At T = 6 months, the other five dogs were euthanized and their IVDs were analyzed.

The first objective of this study was to determine the effect of NCM (produced from porcine NC-rich tissue by lyophilization, pulverization, and resuspension at 10 mg/mL) on human and canine CLCs *in vitro*. Porcine NCM was pooled (*n* = 6) to assess the its effect on a representative population of donor-specific (CLCs from) degenerated IVDs (*n* = 6 canine and *n* = 6 human CLC donors). The second aim was to assess the effect of NCM *in vivo* on degenerated canine IVDs (*n* = 6 Beagles, based on power analysis with power: 85%, alpha: 0.8% and standard deviation 15–30%, with disc height index as the primary read out parameter [29, 44]). Also, the (additive) effect of MSCs was determined. One dog died during the intradiscal injections at T = 0 months (cause of death unrelated to treatment) and therefore, at T = 6 months, the IVDs of the five remaining Beagles were analyzed. We hypothesized that NCM would exert regenerative effects on canine and human CLCs *in vitro* and degenerated canine IVDs *in vivo* and that MSCs would have an additive effect. For the *in vivo* experiment, NCM, MSCs and NCM+MSCs were intradiscally injected in mildly (spontaneously) and moderately (induced by partial NP removal) degenerated canine IVDs (Figure 9). Treatments were not randomized within each dog to prevent interference of random effects from the spinal segment. After three months, NCM was reinjected in two degenerated IVDs per dog (2x NCM) to determine whether multiple injections would exert a more beneficial effect. Longitudinal quantitative MRI was performed and IVDs were macroscopically, histologically, and biochemically analyzed after six months. Outliers were not excluded. The investigators who assessed, measured, or quantified the results were blinded to the intervention.

### Generation of NCM

Thompson score I, healthy IVD tissue was collected from complete spines of six 3-month-old porcine donors from the slaughterhouse in accordance with local regulations (permit 457642.09). To produce NCM, NP tissue was lyophilized overnight, pulverized to fine powder using a microdismembrator (Sartorius) and resuspended at 10 mg/mL in hgDMEM+Glutamax (31966, Invitrogen). For *in vitro* studies, NCM was supplemented with 1% P/S (P11-010, GE Healthcare Life Sciences), 1% ITS+ premix (354352, Corning Life Sciences), 0.04 mg/mL L-proline (P5607, Sigma-Aldrich), 0.1 mM Ascorbic acid 2-phosphate (Asap; A8960, Sigma-Aldrich), and 1.25 mg/mL Bovine Serum Albumin (BSA; A9418, Sigma-Aldrich).

### The effect of NCM on canine and human CLCs and MSCs *in vitro*

IVDs from complete spines were collected from six Beagles (2–7 years of age, three male/three female, Thompson score III) euthanized in unrelated research studies (approved by the Utrecht University Animal Ethics Committee, experimental numbers: 2012.III.07.065, 2013.III.02.017, and 2013.II.12.126). Additionally, IVDs from six human donors (47–72 years of age, three male/three female, Thompson score III) were obtained during a standard *post-mortem* diagnostics (<48 hours after death). The L2-L5 part of the spine was collected, approved by the scientific committee of the Pathology department of the University Medical Centre Utrecht (UMCU). Anonymous use of redundant tissue for research purposes is a standard agreement with UMCU patients (Local Medical Ethical Committee number 12–364). The IVDs were used according to the code ‘Proper Secondary Use of Human Tissue’, installed by the Federation of Biomedical Scientific Societies. The NP was collected by precise separation from AF and EPs and digested by 0.15% pronase (45 min) and 0.15% collagenase (overnight) treatment. The CLCs were stored in hgDMEM+Glutamax with 10% fetal bovine serum and 10% DMSO (−196° C) until use.

Canine and human CLCs were expanded at 5% O_2_, 5% CO_2_, 37° C until passage 2 as described previously [5]. Additionally, bone marrow-derived MSCs from three canine donors (two 4-month old Labrador Retrievers, one 3-year old Beagle, male) were isolated, expanded, and characterized as described previously [45]. Micro-aggregates of 35,000 CLCs or MSCs were formed as described previously [5]. Briefly, CLCs/MSCs were plated in low-adherence cell-repellent surface 96-well plates (650970, CELLSTAR^®^ Greiner Bio-one) in 50 µL basal culture medium (hgDMEM+Glutamax with 1% P/S, 1% ITS+ premix, 0.04 mg/mL L-proline, 0.1 mM Asap, 1.25 mg/mL BSA). CLC micro-aggregates (*n* = 6, in duplicates) were cultured in basal culture medium for (negative) control micro-aggregates, supplemented with 10 ng/mL human recombinant TGF-β_1_ (240-B, R&D Systems) as positive control (to show that the canine and human CLC donors were able to produce GAGs if a proper stimulus was provided, *e.g.* in case these donors would not respond to NCM), or 10 mg/mL NCM. MSC micro-aggregates (*n* = 3, in duplicates) were cultured in (a) basal culture medium supplemented with 0.1 µM dexamethasone (D8893, Sigma Aldrich) (control), (b) supplemented with 10 ng/mL TGF-β_1_ (positive control), or (c) 10 mg/mL NCM. Micro-aggregates were cultured for 28 days at 5% O_2_, 5% CO_2_, 37° C. Culture medium was changed twice weekly.

The DNA (dsDNA High Sensitivity Assay Kit, Invitrogen) and GAG content (DMMB assay [46]) were determined at day 28, and Safranin O/Fast Green staining and immunohistochemistry for collagen type I, II, and X were performed at day 28 (*n* = 6, in duplicates) as described previously [5]. Sulphate incorporation (measure for proteoglycan synthesis rate) was determined during the last 4 hours of a 7-day culture period as described previously [47] (details in Supplementary File 5A).

### Experimental setup of the *in vivo* study

Procedures were approved and conducted in accordance with Animal Experiments Committee guidelines (project number: AVD108002015285), required by Dutch regulation. Six intact female Beagles (14 months of age, weight 10–11 kg) were purchased from Marshall BioResources. Bone marrow-derived MSCs were obtained from one 3-year old male canine donor (Beagle, similar donor as used in *in vitro* experiment, euthanatized for unrelated experiments (2012.III.07.065) to enable tracking of male DNA (MSCs) in female IVDs.

The effect of NCM, MSCs and a combined treatment of NCM+MSCs was tested on mildly and moderately degenerated canine IVDs (Figure 9). Six weeks before the intradiscal injections (T = −1.5 months), dogs underwent a general, orthopedic and neurologic clinical examination by veterinarians (FB, AT). Additionally, more severe (moderate) IVD degeneration was induced in five IVDs per dog by a board-certified veterinary surgeon (BM) via partial NP removal (NX) on the left side of the spine. At T = 0 months, MRI analysis was performed to determine the degree of IVD degeneration. Thereafter, the IVDs in which no NX was performed (noNX-IVDs; five per dog) and the IVDs in which NX was performed (NX-IVDs; five per dog) were either not injected (control) or injected on the right side of the spine with 50 µL of (a) 1 × 10^6^ MSCs in a canine albumin-hyaluronan hydrogel [48] (b) 10 mg/mL NCM, or (c) 1 × 10^6^ MSCs suspended in 10 mg/mL NCM. At T = 3 months, MRI analysis was performed and two IVDs per dog (L6-L7 (noNX-IVD) and L7-S1 (NX-IVD), which previously received 50 µL NCM) were reinjected with 50 µL of 10 mg/mL NCM (2x NCM), to test whether a better effect could be achieved with multiple injections. At T = 6 months, MRI and CT analysis were performed, the dogs were euthanized, and the IVDs were collected. One dog died at T = 0 months during the first injection (cause of death unrelated with treatment). This dog was used to determine baseline degree of induced IVD degeneration and confirm the presence of transplanted MSCs.

### Induction of IVD degeneration

Anesthesia and analgesia protocols are described in Supplementary File 5B. IVD degeneration was induced either by a left lateral (T11-T12 until L5-L6) or a dorsal (L6-L7 and L7-S1, assisted by mini-laminectomy) nuclectomy under fluoroscopic guidance by a board-certified veterinary surgeon (BM). A 2 mm slit was made in the AF with a Beaver knife 65 and NP tissue (20–40 mg, dependent on IVD size) was collected using a 1 mm ball-tipped probe, and fixed in 4% neutral buffered formaldehyde. Five μm paraffin sections were stained with Hematoxylin (109249, Merck)/Eosin (115935, Merck) and Picrosirius Red (saturated aqueous picric acid: P6744, Sigma-Aldrich; sirius red: 365548, Sigma-Aldrich)/Alcian Blue (A5268, Sigma-Aldrich) [49]. All dogs uneventfully recovered from the induction surgery and were ambulant the next day.

### Preparation of MSCs for *in vivo* application

Chondrogenic (Safranin O/Fast Green), adipogenic (Oil-Red-O), osteogenic (Alizarin Red S staining) differentiation and FACS analysis for positive (CD29 (303004, Biolegend), CD90 (12590042, Thermofisher scientific), CD105 (bs-4609R, Bioss antibodies)) and negative (CD34 (559369, BD Pharmingen), CD45 (LS-C127720-100, LifeSpan BioSciences)) MSC markers [50, 51] was performed as described previously [45]. MSCs from the Beagle donor were expanded until passage 1 and incorporated in (a) albumin-hyaluronan hydrogels (20 × 10^6^ MSCs/mL hydrogel) or (b) 10 mg/mL NCM (20 × 10^6^ MSCs/mL NCM) directly prior to injection. The hydrogels were composed of chemically activated canine albumin (Animal Blood Resources International), bisthio-polyethylene glycol, and hyaluronic acid.

### Intradiscal injections

Intradiscal injections of T12-13 until L5-L6 were performed from the right side with 25G needles (Epican, 4502400, B.Braun). The L6-L7 and L7-S1 IVDs were percutaneously approached dorsally with 19G needles (301750, BD Microlance) that guided the 25G needles into the NP under fluoroscopic guidance (Supplementary File 2G–2H). Once the needle was correctly placed according to fluoroscopic imaging, 50 µL of the treatment volume was intradiscally administered by a board-certified veterinary surgeon (BM) under visual control of the injection port. Leakage through the injection port was not observed. One dog died unexpectedly at T = 0 (cause of death unrelated to treatment) and the IVDs of this dog were used as baseline. The five other dogs uneventfully recovered from the injections and were ambulant the next day. One dog showed minor paresis and a reduction in spinal reflexes of the right hind limb related to the surgical approach, but recovered within seven days.

### MRI and CT

Details for the magnetic resonance images (MRI) and computed tomography (CT) analysis are given in Supplementary File 5C. MRI T2 mapping and T1ρ values were computed by voxelwise fitting. The mean signal intensity in each region of interest was calculated using the Levenberg-Marquardt nonlinear least-squares method as described previously [52]. MRI images were blindly evaluated by two independent investigators (FB, MB; Pfirrmann score) [22]. The Pfirrmann score (grade 1–5; grade 1 represents a healthy and grade 5 a severely degenerated IVD) is used to determine the degree of IVD degeneration, taking into account the structure and signal intensity of the NP, the distinction between NP and AF border, and the height of the IVD [22]. The Pfirrmann, and T1ρ and T2 mapping inter- and intra-observer reliability was excellent (intra-class correlation >0.95) [53]. Disc height index was calculated on T2W images according to the method described by Masuda *et al.* [54]. In short, the DHI was calculated by averaging the widths of the dorsal, middle, and ventral parts of the vertebral disc divided by the average of dorsal, middle, and ventral body heights of the adjacent cranial and caudal vertebrae.

### Sample collection, macroscopic and histopathological grading

Details for samples collection are provided in Supplementary File 5D. IVD images were blindly evaluated by two independent investigators (FB, AT; Thompson score) [21]. The Thomson score (grade 1–5; grade 1 represents a healthy and grade 5 a severely degenerated IVD) is used to macroscopically determine the degree of IVD degeneration and includes all IVD units (AF, NP, EP and vertebrae) [21]. Inter-observer reliability was excellent (intra-class correlation: 0.85) [53]. Five μm sections were stained with H/E and Picrosirius Red/Alcian Blue (PSR/AB) and blindly evaluated (modified Boos score) [23]. The modified Boos score (0–29) is used to determine the degree of histologic canine IVD degeneration and includes the following subcriteria: morphology of the AF, chondrocyte metaplasia in the AF, tear and cleft formation (in NP and AF), chondrocyte proliferation in the NP, presence of NCs in the NP, PSR/AB matrix staining of the NP, EP morphology, new bone formation, and subchondral bone sclerosis [23]. Immunohistochemistry for collagen type I, II, and X [5] and COX-2 [55] were performed as described previously. For COX-2, (positively stained) cell numbers in four randomly selected NP areas per IVD section were manually counted (Adobe Photoshop CC). The mean percentage of immunopositive cells was calculated.

### Gene expression profiling and biochemical analysis

Details on RNA isolation, cDNA synthesis, and RT-qPCR are provided in Supplementary File 5E. To determine the GAG and DNA content, the NP and AF samples were homogenized in complete lysis buffer using TissueLyser II (Qiagen) for 4 minutes at 20 Hz. PGE2 levels were determined in the supernatant using a colorimetric competitive enzyme immunoassay kit (PGE2 high sensitivity EIA kit, ENZO Life Sciences) as described previously [55]. The DNA and GAG content were measured in papain-digested supernatant and pellet as described previously [52]. A hydroxyproline assay [56] was used to determine the samples collagen content as described previously [52]. Briefly, samples were freeze-dried overnight, hydrolyzed in 4 M NaOH at 108° C overnight, centrifuged (15 seconds, 14,000 *g*) and stored at −20° C. Prior to measurement, samples were centrifuged (15 seconds, 14,000 *g*), chloramine T reagent (2426, Merck) was added, and samples were shaken for 20 minutes (170 rpm). Freshly prepared dimethylaminobenzaldehyde (3058, Merck) was added, samples were incubated for 20 minutes at 60° C, the absorbance was read (570 nm), and the collagen content was calculated by multiplying the hydroxyproline content with factor 7.5 [52]. MSC fate was determined by *SRY:GAPDH* PCR on genomic DNA isolated from papain-digested sample as described previously [57]. The detection limit of this assay was 250 MSCs/µL buffer (determined by a dilution series of positive control sample), which equals 25,000 MSCs/Beagle NP.

### Statistical analyses

Statistical analyses were performed using IBM SPSS Statistics 24 and R studio. The Shapiro Wilks test was used to examine the data for normal distribution. Non-parametric data were converted to normally distributed data (log transformation) when possible. Two-sided testing was performed. To correct for multiple comparisons, Benjamini & Hochberg False Discovery Rate *post-hoc* tests were performed. For *in vitro* experiments, normally distributed data were examined using general linear regression models based on ANOVAs, whereas non-normally distributed data were subjected to Kruskal Wallis and Mann–Whitney *U* tests. For *in vivo* experiments, a randomized block design ANOVA was used for normally distributed data (DNA, GAG, collagen, PGE2 content). For DHI, quantitative T1ρ and T2 mapping, and RT-qPCR data, Cox proportional hazard tests were performed (random effect: donor). For Boos, Thompson, and Pfirrmann data analysis, Mann–Whitney Wilcoxon tests were performed. Because of the small *in vivo* sample size, for all comparisons with 0.05 ≥ *p* ≤ 0.20, effect sizes (ES; Hedge’s g for normally distributed data, Cliff’s delta for non-parametric data) were determined and classified as described previously [58, 59]. *p*-values < 0.05 were considered significant. Medium-very large effect sizes (Hedge’s g ≥ 0.5, Cliff’s delta ≥ 0.28) were considered relevant.

## CONCLUSIONS

The present study demonstrates that NCM exerted regenerative effects on canine MSCs and on canine and human CLCs from degenerated IVDs *in vitro* as well as on canine IVDs *in vivo*. *In vivo*, NCM mainly exerted effects on moderately degenerated IVDs; a repeated intradiscal NCM injection exerted beneficial effects on macroscopic, radiologic, biochemical, and histological level and inhibited inflammation. This is the first study that shows that intradiscally injected NCM could potentially be a promising treatment for human and canine IVD disease, by harnessing the NC regenerative and anti-inflammatory potential, and circumventing the challenging identification of bioactive NC-secreted factors. This approach should be feasible in light of the wide clinical application of demineralized bone matrix within the bone regeneration field. Future studies should focus on removal of nucleic acid from NCM and the mechanism of NCM-mediated regeneration.

## SUPPLEMENTARY MATERIALS FIGURES AND TABLE



## Abbreviations

ACAN: aggrecan
ADAMTS5: a disintegrin and metalloproteinase with thrombospondin motifs 5
AF: annulus fibrosus
AGE: advanced glycation end products
BAX: B-cell lymphoma 2 associated X
BCL2: B-cell lymphoma 2
CASP3: caspase 3
CCND1: cyclin D1
CLC: chondrocyte-like cell
COL1A1: collagen type 1A1
COL2A1: collagen type 2A1
COL10A1: collagen type 10A1
COX-2: cyclo-oxygenase-2
CT: computer tomography
DHI: disc height index
DMB: demineralized bone matrix
ECM: extracellular matrix
EP: end plate
ES: effect size
GAG: glycosaminoglycan
IL-1β: interleukin 1β
IVD: intervertebral disc
LBP: low back pain
MC: modic change
MMP13: martix metalloproteinase 13
MRI: magnetic resonance imaging
MSC: mesenchymal stromal cell
NC: notochordal cell
NCM: notochordal cell-derived matrix
NP: nucleus pulposus
NX: partial nucleus pulposus removal
PAI1: plasminogen activator inhibitor type 1
PGE2: prostaglandin E2
SOX9: sex-determining region of Y-box9
SRY: sex-determining region of Y
TGF-β: transforming growth factor-β
TIMP1: tissue inhibitor of metalloproteinases
TNFα: tumor necrosis factor α
