# Supplementary material for: Biologic canine and human intervertebral disc repair by notochordal cell-derived matrix: from bench towards bedside
Source: Oncotarget. 2018 May 29;9(41):26507–26. doi: 10.18632/oncotarget.25476 (PMC5995168; doi:10.18632/oncotarget.25476)
Supplement: Supplementary file 1 [file oncotarget-09-26507-s001.pdf]

Biologic canine and human intervertebral disc repair by notochordal cell-derived matrix: from bench towards bedside

SUPPLEMENTARY MATERIALS

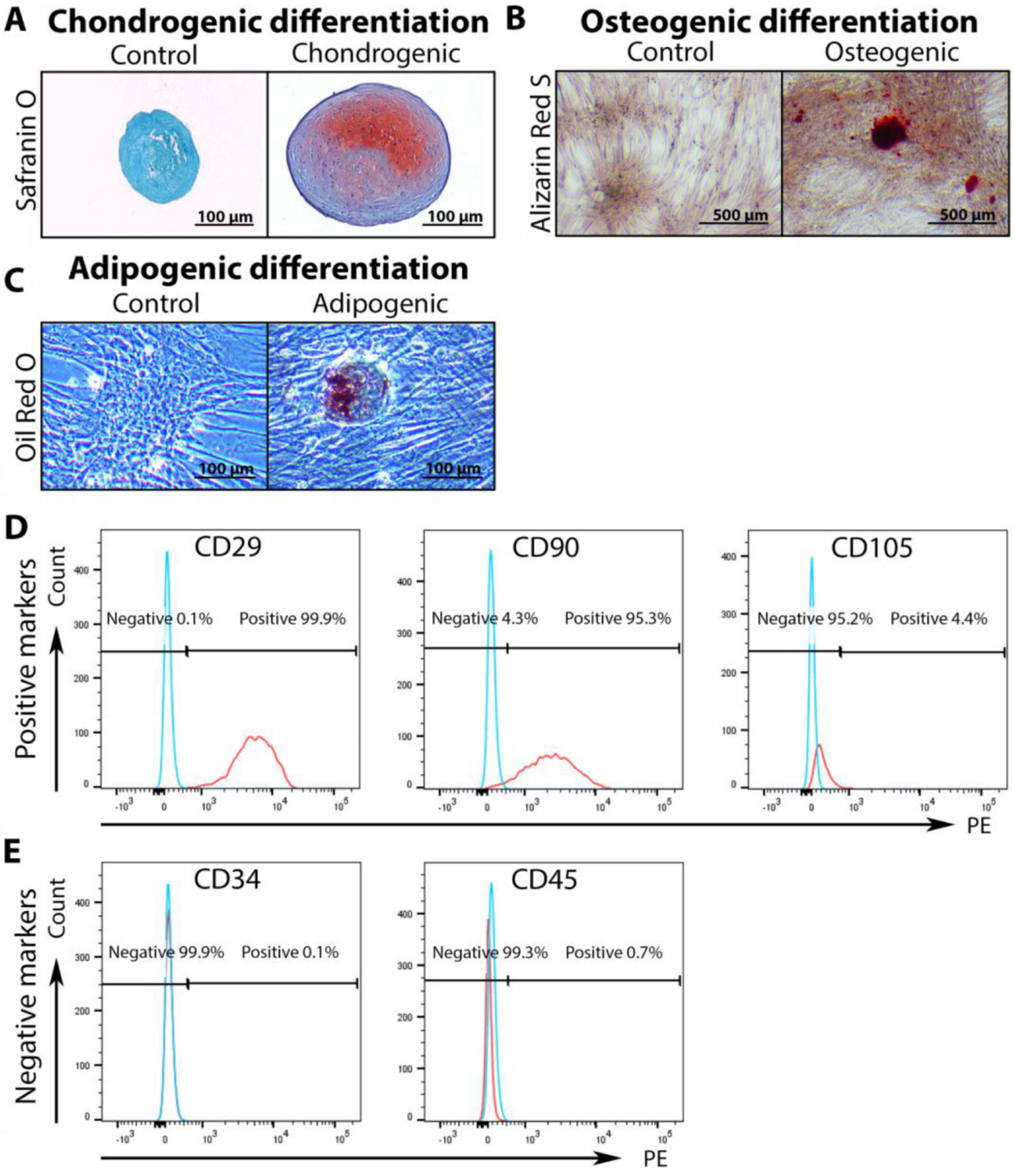

Supplementary File 1: Mesenchymal stromal cell (MSC) characterisation. Characterisation of bone marrow-derived MSCs of the male Beagle donor used in the *in vivo* experiment. (**A**) Chondrogenic (Safranin O/Fast Green staining), (**B**) osteogenic (Alizarin Red S staining), and (**C**) adipogenic (Oil-Red-O staining) differentiation and (**D**) FACS analysis for positive (CD29, CD90, CD105) and (**E**) negative (CD34, CD45) MSC markers. Chondrogenic, osteogenic, and adipogenic differentiation was successfully achieved. The cells were found to be $CD34^-$, $CD45^-$, $CD29^+$ and $CD90^+$. However, only 4.4% of cells was $CD105^+$, this could be explained by a low cross-species reactivity of the anti-human CD105, which was used due to unavailability of commercial anti-canine CD105. Altogether, the results confirm the presence of MSCs in the injected cells. PE: Phycoerythrin fluorescent dye.

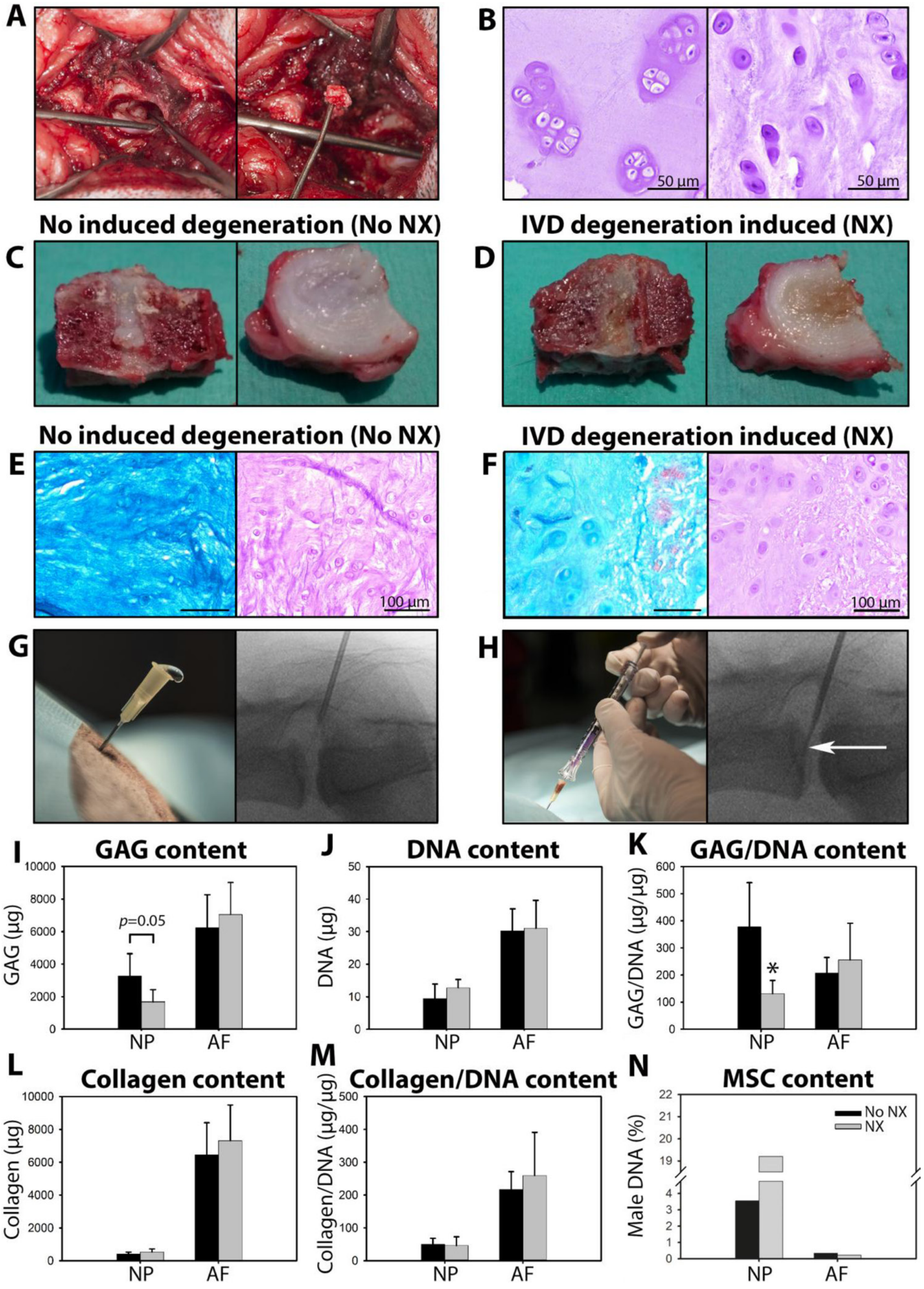

Supplementary File 2: Induction of intervertebral disc (IVD) degeneration by partial nucleus pulposus (NP) removal. Six weeks before the start of the experiment (T = −1.5 months), moderate IVD degeneration was induced in five IVDs per dog by partial removal of the NP (NX; T12-T13, L1-L2, L3-L4, L5-L6, and L7-S1) (**A**). The removed NP tissue showed chondrocyte-like cell (CLC) clusters and single cells (H&E staining) (**B**). At the start of the experiment (T = 0 months), the IVDs of the dog that died unexpectedly were used for baseline analysis and were examined macroscopically (**C**, **D**), histologically (Alcian Blue-Picrosirius Red (left) and H&E staining (right) (NP tissue with CLCs) (**E**, **F**)) and biochemically for glycosaminoglycan (GAG; **I**), DNA (**J**), GAG/DNA (K), collagen (**L**) and collagen/DNA (**M**) content. The NPs of the IVDs in which moderate degeneration was induced by NX showed macroscopically a brown discoloration, histologically less blue (GAG) and more red (collagen) in their NP tissue, and biochemically a decreased GAG and GAG/DNA content. Pictures of the (fluoroscopic) through the needle injections with NCM/mesenchymal stromal cells (MSCs) are shown in (**G**) (positioning of the 19G needle) and (**H**) (through the needle injection with a 25 G needle, arrow). The fate of the MSCs (at T = 0 months, directly after injection) was determined by *SRY:GAPDH* PCR on genomic DNA of the deceased dog IVDs (N). The male DNA percentage in the female IVD indicates the percentage of MSCs present in the IVD. *n* = 5 IVDs per condition (DNA, GAG, collagen content) and *n* = 1 IVD per condition (MSC content, male DNA percentage).

Supplementary File 3: Modic changes, periosteal bone formation, spondylosis and end plate (EP) lysis in the intervertebral discs (IVDs).

The development of Modic Changes (MC) was recorded during the study. In noNX-IVDs, only one MC type 3 was observed at T = 0, 3, and 6 months. In NX-IVDs, one and a half month after NX, and before the different treatments were applied (T = 0 months), several MCs were already present. During the study, i.e. after the first intradiscal injections, control NX-IVDs developed two type 1 MCs, while 1xNCM-treated NX-IVDs developed one additional type 1 MC and one type 1 MC progressed towards a type 3 MC, MSC-treated NX-IVDs developed one type 1 MC, and NCM+MSC-treated NX-IVDs developed four type 1 and two type 3 MCs. In contrast, 2xNCM-treated NX-IVDs did not develop any additional MCs, presumably (also) due to the less invasive percutaneous approach of the L7-S1 IVD.

Modic changes at T=0 months

	No NX					NX				
**Dog**	**Control**	**1xNCM**	**2xNCM**	**MSC**	**NCM+MSC**	**Control**	**1xNCM**	**2xNCM**	**MSC**	**NCM+MSC**
**1**	-	-	3	-	-	-	-	-	-	-
**2**	-	-	-	-	-	-	-	-	-	-
**3**	-	-	-	-	-	1	1	-	-	-
**4**	-	-	-	-	-	-	1	2+3	-	-
**5**	-	-	-	-	-	-	-	1+3	1	-

Modic changes at T=3 months

	No NX					NX				
**Dog**	**Control**	**1xNCM**	**2xNCM**	**MSC**	**NCM+MSC**	**Control**	**1xNCM**	**2xNCM**	**MSC**	**NCM+MSC**
**1**	-	-	3	-	-	-	-	-	-	-
**2**	-	-	-	-	-	-	-	-	1	-
**3**	-	-	-	-	-	1	1	-	-	1
**4**	-	-	-	-	-	1	1	2+3	-	1
**5**	-	-	-	-	-	-	1	3	1	1

Modic changes at T=6 months

	No NX					NX				
**Dog**	**Control**	**1xNCM**	**2xNCM**	**MSC**	**NCM+MSC**	**Control**	**1xNCM**	**2xNCM**	**MSC**	**NCM+MSC**
**1**	-	-	3	-	-	-	-	-	-	1
**2**	-	-	-	-	-	-	-	-	1	3
**3**	-	-	-	-	-	1	1	-	-	1
**4**	-	-	-	-	-	-	3	2+3	-	3
**5**	-	-	-	-	-	1	-	1+3	1	-

Overview of the type of Modic changes observed by MRI analysis. - : no Modic changes observed. Red depicted Modic changes are different from T = 0 months (1.5 month after moderate degeneration was induced by partial NP removal (NX)). *n* = 5.

The development of periosteal bone formation and ventral spondylosis was determined at T = 6 months using CT. When these features were present, they were located at the left side of the spinal column, the side employed to induce IVD degeneration. No periosteal bone formation or ventral spondylosis was detected in control, 2xNCM and MSC-treated noNX-IVDs. In contrast, mild ventral spondylosis was observed in 1xNCM-treated (3/5) and NCM+MSC-treated (1/5) noNX-IVDs. Additionally, minimal (1/5) and distinct (1/5) periosteal bone formation was observed in NCM+MSC-treated noNX-IVDs. In NX-IVDs, more pathological features were detected. In 4/5 control NX-IVDs, distinct periosteal bone formation was detected. Furthermore, 1xNCM-treated NX-IVDs showed mild-distinct bone formation (5/5) and mild spondylosis (3/5), and MSC- and MSC+NCM-treated NX-IVDs showed distinct bone formation (2/5 and 4/5, respectively). 2xNCM-treated NX-IVDs showed none of these pathological processes on CT analysis, again presumably due to the less invasive approach of the L7-S1 IVD.

	No NX					NX				
**Dog**	**Control**	**1xNCM**	**2xNCM**	**MSC**	**NCM+MSC**	**Control**	**1xNCM**	**2xNCM**	**MSC**	**NCM+MSC**
**1**	-	- [a]	-	-	-	-	+	-	+	+
**2**	-	-	-	-	-	+	+/- [a]	-	-	-
**3**	-	- [a]	-	-	+/- [a]	+	+	-	-	+
**4**	-	- [a]	-	-	-	+	+ [a]	-	+	+
**5**	-	-	-	-	+	+	+ [a]	-	-	+

- =: no periosteal bone formation, +/-: minimal periosteal bone formation, +: distinct periosteal bone formation.
a: mild ventral spondylosis. Bone formation was only detected at the left side (induction of degeneration side).
n=5

EP lysis (determined by CT analysis at T = 6 months) was only present in NX-IVDs, most probably due to the NX procedure.

	No NX					NX				
**Dog**	**Control**	**1xNCM**	**2xNCM**	**MSC**	**NCM+MSC**	**Control**	**1xNCM**	**2xNCM**	**MSC**	**NCM+MSC**
**1**	-	-	-	-	-	-	-	-	+	-
**2**	-	-	-	-	-	-	+	-	-	+
**3**	-	-	-	-	-	-	-	-	-	+
**4**	-	-	-	-	-	-	+	-	+	+
**5**	-	-	-	-	-	-	-	-	-	+

- =: no EP lysis, +: EP lysis. *n*=5

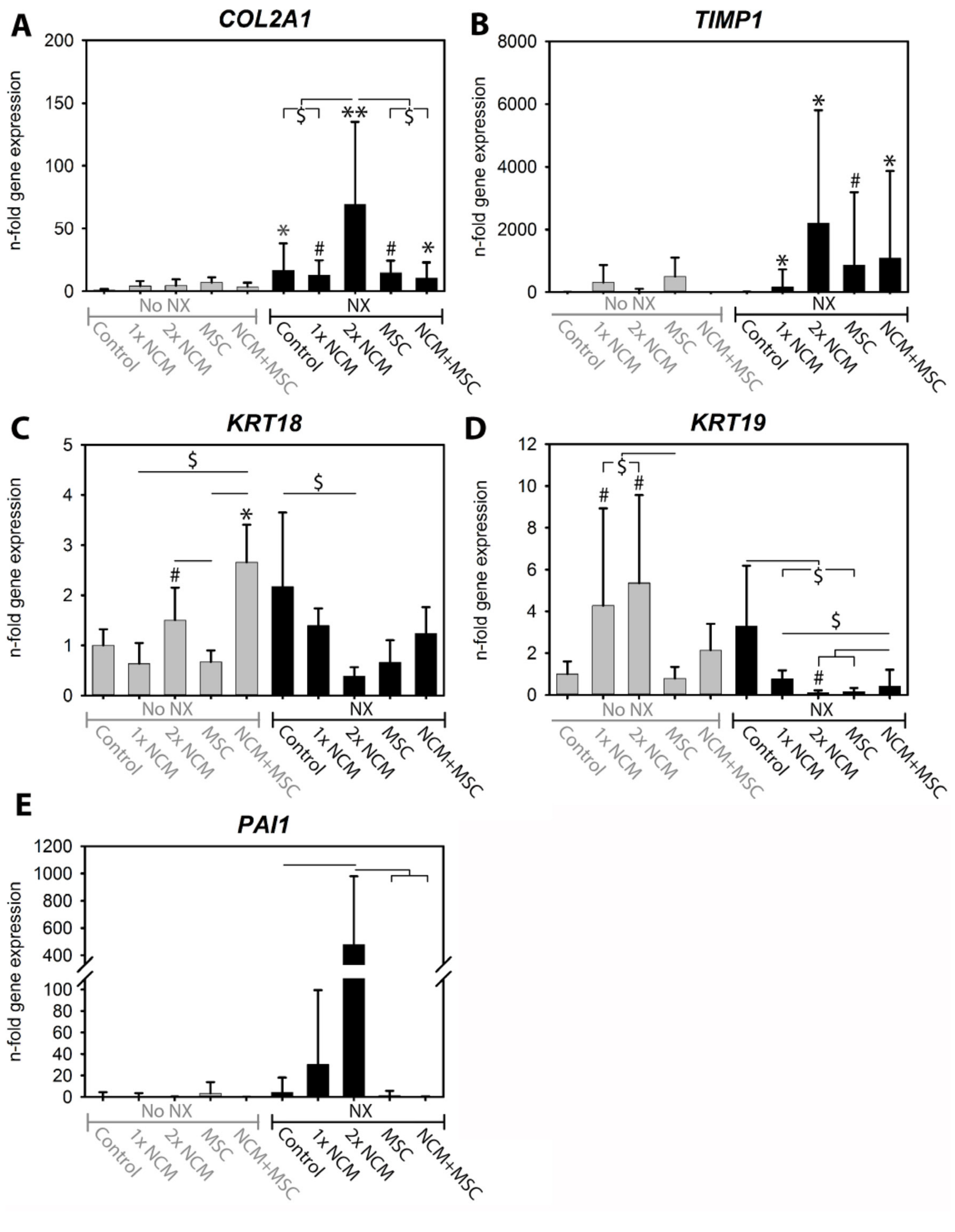

Supplementary File 4: mRNA expression of the degenerated canine intervertebral discs (IVDs) *in vivo*. Relative target gene expression (mean ± SD) of (**A**) *COL2A1*, (**B**), *TIMP1*, (**C**) *KRT18*, (**D**) *KRT19*, and (**E**) *PAI1* at T = 6 months. The control noNX-IVDs were set at 1. NX: partial nucleus pulposus (NP) removal was performed to induce moderate IVD degeneration. Bars represent significant differences between conditions ($p < 0.05$); n.s.: not significantly affected; *,**: significantly different from control noNX-IVDs ($p < 0.05$, $p < 0.01$, respectively); $^{\#}$: different from control noNX-IVDs ($0.05 \geq p \leq 0.20$, medium/large effect size); $^{\$}$: difference between conditions with medium/large effect size ($0.05 \geq p \leq 0.20$); $n = 5$. Data were analyzed with a cox proportional hazard model (donor as random effect). Benjamini & Hochberg False Discovery Rate post-hoc tests were performed to correct for multiple comparisons.

Supplementary File 5: Detailed information on Materials and Methods

I. *In vitro* studies

a. Sulphate incorporation assay

Shortly, chondrocyte-like cells (CLCs) from the six canine and human donors were cultured in micro-aggregates of 35,000 cells in duplicates in basal culture medium, basal culture medium supplemented with 10 ng/mL TGF-β_1 (positive control) or 10 mg/mL NCM. After 7 days, the micro-aggregates were pulsed with 20 mCi $^{35}SO_4^{2-}$. Four hours later, the micro-aggregates were washed twice with 500 µL PBS and frozen at −20° C. After one day, the micro-aggregates were digested for two hours in papain buffer. The next day, the amount of $^{35}SO_4^{2}$-labelled GAGs in the papain digest was measured by liquid scintillation analysis and normalized for the micro-aggregates' DNA content (measured using the dsDNA High Sensitivity Assay Kit, Invitrogen).

II. *In vivo* studies

b. Anesthesia and analgesia protocols Beagles *in vivo*

For the induction of degeneration and intradiscal injections anesthesia was provided by intravenous buprenorphine (20 µg/kg) and dexmedetomedine (10 µg/kg), induced by intravenous propofol (1–2 mg/kg) and maintained by intravenous propofol (100 mg/kg/hr) and inhalation of 1–1.5% isoflurane. Peri- and postoperative analgesia was provided by intramuscular buprenorphine (20 µg/kg, one day, three times a day) and subcutaneous carprofen (4 mg/kg, five days, once a day). Additionally, the dogs received preoperative intravenous cefazolin (20 mg/kg) once. For MRI and CT, anesthesia was provided by intravenous butorphanol (0.2 mg/kg) and dexmedetomedine (10 µg/kg), induced by intravenous propofol (1–2 mg/kg) and maintained by inhalation of 1–1.5% isoflurane. At T = 6 months, the Beagles were euthanized after the MRI by intravenous pentobarbital (200 mg/kg).

c. MRI and CT

Magnetic resonance images (MRI) were obtained at T = 0, 3, and 6 months using a 1.5 T high field MRI unit (Ingenia, Philips). Sagittal T1-weighted Turbo Spin Echo (repetition time (TR): 400 ms, echo time (TE): 8 ms), and T2-weighted Turbo Spin Echo (TR: 3000 ms, TE: 110 ms) images were acquired using a field of view (FOV) of 75 × 220 mm and acquisition matrix of 124 × 313 and 124 × 261. Thirteen 2 mm-thick slices covered the spine from T10 to S1. For T2 mapping, a quantitative multiple spin-echo T2-mapping sequence was used with the following parameters; FOV: 75 × 219 mm, acquisition matrix: 96 × 273, slice thickness: 3 mm, TR: 2000 ms. Eight echoes were acquired with TE: 13–104 ms with 13 ms echo spacing. Sagittal T1ρ-weighted imaging was performed using a spinlock-prepared sequence with 3D multi-shot gradient echo (T1-TFE) readout with FOV: 76 × 220 mm, acquisition matrix: 76 × 220, slice thickness: 2 mm, TR/TE: 4.6 s/2.3 s, TR: 5ms, TE: 2.5 ms, TFE factor: 50, flip angle: 45°, shot interval: 3000 ms. For quantitative T1ρ mapping, data were acquired with spinlock times: 0, 10, 20, 30 and 40 ms, spinlock pulse amplitude: 500 Hz. An oval region of interest (ROI) was manually segmented on the NP of all spinal segments.

Computed tomography (CT) images were obtained *postmortem* with dogs positioned in dorsal recumbency to monitor extradiscal calcification and EP pathology. The CT scans were made using a 64 slice CT scanner (Siemens Somatom Definition AS, Siemens Healthcare) with the following parameters: 120 kV, 350 mas, 1000 ms tube rotation time, 0.6 mm slice thickness, 0.35 spiral pitch factor, 512 × 512 pixel matrix and 93 mm fixed field view. Reconstructions were made in transverse and sagittal planes using soft tissue and bone reconstruction kernels and images were reviewed in soft tissue/bone settings (window length 50, width 300, and window length 600, width 3000, respectively).

d. Sample collection

The vertebral column (T11-S1) was harvested using an electric multipurpose saw (Bosch). The muscles were removed and the vertebrae were transversely transected using a band saw (EXAKT tape saw). The ten spinal units (½ vertebra – endplate - IVD - endplate - ½ vertebra) were sagitally transected into two identical parts using a diamond band saw (EXAKT 312). In one part the bisected IVD tissue was isolated from the endplate and vertebra with a surgical knife and was snap frozen in liquid nitrogen and stored at −70° C for biochemical analyses. The other part was used to photograph (Olympus VR-340) the other half of the IVD for macroscopic Thompson score evaluation. The samples were fixed in 4% buffered formaldehyde for 14 days, decalcified in PBS with 0.5M EDTA for two months and embedded in paraffin.

e. RNA isolation, cDNA synthesis, and RT-qPCR

For RT-qPCR, the NP and AF were carefully separated and cut into small pieces. Half of the NP/AF tissues was collected in 400 and 750 µL complete lysis buffer (04719956001, Roche Diagnostics), respectively, and stored at −70° C until

biochemical analysis. The other tissue halves were separately collected in 300 µL Exiqon Kit RNA buffer and stored at −70° C until biomolecular analysis. The RNA samples were homogenized using the TissueLyser II (Qiagen) for 4 minutes at 20 Hz. Thereafter, RNA was isolated (miRCURY RNA Isolation Kit (300110, Exiqon)) and cDNA was synthesized (iScriptTM cDNA Synthesis Kit (170-8891, Bio-Rad)) and amplified (Sso AdvancedTM PreAmp Supermix (1725160, Bio-Rad)) according to the manufacturer's instructions. RT-qPCR was performed as described earlier [1] with maximal RNA input. Briefly, RT-qPCR was performed using the iQT™ SYBR Green Supermix Kit and the CFX384 Touch™ Real-Time PCR Detection System (Bio-Rad). For determination of relative gene expression, the Normfirst ($E^{\Delta\Delta}Cq$) method was used. For every target gene, the mean n-fold changes and standard deviations in gene expression were calculated. Four stably expressed reference genes were chosen to normalize target gene expression.

Primers used for quantitative RT-qPCR of canine samples

Genes	Forward sequence 5′ → 3′	Reverse sequence 5′ → 3′	Amplicon size	Annealing temp (°C)
**Reference genes**				
GAPDH	TGTCCCCACCCCCAATGTATC	CTCCGATGCCTGCTTCACTACCTT	100	58
HPRT	AGCTTGCTGGTGAAAAGGAC	TTATAGTCAAGGGCATATCC	104	58
RPS19	CCTTCCTCAAAAAGTCTGGG	GTTCTCATCGTAGGGAGCAAG	95	61
SDHA	GCCTTGGATCTCTTGATGGA	TTCTTGGCTCTTATGCGATG	92	56.5
**Target genes**				
ACAN	GGACACTCCTTGCAATTTGAG	GTCATTCCACTCTCCCTTCTC	111	62
ADAMTS5	CTACTGCACAGGGAAGAG	GAACCCATTCCACAAATGTC	149	61
CASP3	CGGACTTCTTGTATGCTTACTC	CACAAAGTGACTGGATGAACC	89	61
CCND1	GCCTCGAAGATGAAGGAGAC	CAGTTTGTTCACCAGGAGCA	117	60
COL1A1	GTGTGTACAGAACGGCCTCA	TCGCAAATCACGTCATCG	109	61
COL2A1	GCAGCAAGAGCAAGGAC	TTCTGAGAGCCCTCGGT	151	62
COL10A1	CCAACACCAAGACACAG	CAGGAATACCTTGCTCTC	80	61
IL-1ß	TGCTGCCAAGACCTGAACCAC	TCCAAAGCTACAATGACTGACACG	115	68
KRT18	GGACAGCTCTGACTCCAGGT	AGCTTGGAGAACAGCCTGAG	97	60
KRT19	GCCCAGCTGAGCGATGTGC	TGCTCCAGCCGTGACTTGATGT	86	64
MMP13	CTGAGGAAGACTTCCAGCTT	TTGGACCACTTGAGAGTTCG	250	65
PAI1	AAACCTGGCGGACTTCTC	ACTGTGCCACTCTCATTCAC	98	61.5
SOX9	CGCTCGCAGTACGACTACAC	GGGGTTCATGTAGGTGAAGG	105	62
TIMP1	GGCGTTATGAGATCAAGATGAC	ACCTGTGCAAGTATCCGC	120	66
TNFa	CCCCGGGCTCCAGAAGGTG	GCAGCAGGCAGAAGAGTGTGGTG		65

Reference genes: *HPRT*: Hypoxanthine-guanine phosphoribosyltransferase, *SDHA*: Succinate dehydrogenase complex subunit A, *TBP*: TATA-Box binding protein, *YWHAZ*: tyrosine 3-monooxygenase/tryptophan 5-monooxygenase activation protein zeta, *RPS19*: 40S ribosomal protein S19.
Target genes: *ACAN*: aggrecan, *ADAMTS5*: A disintegrin and metalloproteinase with thrombospondin motifs 5, *CASP3*: caspase 3, *CCND1:* cyclin D1, *COL1A1*: collagen type I, *COL2A1*: collagen type II, *COL10A1*: collagen type X, *IL-1ß*: interleukin-1β, *KRT18*: cytokeratin 18, *KRT19*: cytokeratin 19, *MMP13*: matrix metalloproteinase 13, *PAI1*: Plasminogen activator inhibitor type 1, *SOX9*: sex determining region Y-box 9, *TIMP1*: tissue inhibitor of metalloproteinases, *TNFα*: Tumor Necrosis Factor alpha.
